# Fibroblast Growth Factor 21 Predicts Short-Term Prognosis in Patients With Acute Heart Failure: A Prospective Cohort Study

**DOI:** 10.3389/fcvm.2022.834967

**Published:** 2022-03-16

**Authors:** Guihai Wu, Shenglin Wu, Jingyi Yan, Shanshan Gao, Jinxiu Zhu, Minghui Yue, Zexin Li, Xuerui Tan

**Affiliations:** ^1^Department of Cardiovascular Medicine, First Affiliated Hospital of Shantou University Medical College, Shantou, China; ^2^Clinical Research Center, The First Affiliated Hospital of Shantou University Medical College, Shantou, China

**Keywords:** FGF21, acute heart failure, biomarker, prognosis, death

## Abstract

**Background:**

Recent studies of fibroblast growth factor 21 (FGF21), first recognized as a regulator of glucose and lipid metabolism, have found that the level of in serum FGF21 is associated with the prognosis of many cardiovascular diseases, but its relationship to acute heart failure (AHF) patients remains unknown. Our study aimed to investigate whether circulating FGF21 could predict the short-term prognosis of AHF patients.

**Methods:**

Four hundred and two AHF patients and 19 healthy controls were recruited into the prospective cohort study, and blood samples of participants were collected, in tubes without anticoagulant, within the first 24 h after hospital admission. Serum FGF21 levels were detected by enzyme-linked immunosorbent assay (ELISA). All patients were followed-up at least 6 months after discharge. The primary endpoint was all-cause death, and secondary endpoint was a composite endpoint of death and heart failure readmission. Mortality and composite end point events were analyzed using Kaplan-Meier curves. ROC curves compared the difference between the FGF21 and NT-proBNP in predicting 3- and 6-months mortality. Time-to-event data were evaluated using Kaplan-Meier estimation and Cox proportional hazards models.

**Results:**

In the present study, the serum FGF21 concentrations were significantly higher in the 402 AHF patients enrolled, compared with the 19 healthy controls (*p* < 0.001). The average age was 70 (±12) years, and 58% were males. Participants were divided into two groups according to the median FGF21 level (262 pg/ml): a high FGF21 group (*n* = 201, FGF21 ≥ 262 pg/ml) and low FGF21 group (*n* = 201, FGF21 <262 pg/ml). FGF21 was positively correlated with NT-proBNP, BUN, AST, creatinine and cholesterol, and negatively correlated with ALB and HDL. After a median follow-up of 193 days, the high FGF21 group had higher mortality and composite endpoint events compared with the low FGF21 group (HR: 3.91, 95% CI 2.21–6.92, p <0.001), even after adjusting for NT-proBNP (HR: 3.17, 95% CI 1.72–5.81, *p* < 0.001). ROC analysis shows that FGF21 was better than NT-proBNP in predicting death at both 3 (AUC, 0.77 vs. 0.63, *p* < 0.001) and 6 months (AUC, 0.78 vs. 0.66).

**Conclusion:**

High baseline FGF21 levels are associated with adverse clinical outcomes in AHF patients. Serum FGF21 might be a potential predictive biomarker of AHF patients.

## Introduction

Previous studies indicate that mortality rates in AHF patients range from 18% to 33% after discharge (1, 2). It is important for clinicians to estimate the state of AHF patients and provide timely treatment measures. Biomarkers can reflect the changes of pathological mechanisms in AHF patients, and so reflect the severity of the disease and prognosis of patients. At present, natriuretic peptides (BNP and NT-ProBNP) are clinically regarded as important markers for the diagnosis of AHF, and a role in prognosis has also been identified (3). Natriuretic peptide is secreted into the blood when the cardiac volume load is increased, so it mainly reflects the condition of the heart. Multiple organ failure syndrome, commonly of the liver and kidneys, is one of the characteristics of AHF, and the degree of liver and renal function damage is closely related to prognosis (4, 5). Therefore, natriuretic peptide as a prognostic marker in AHF is insufficient. Thus, new biomarkers are needed to improve the prognostic prediction of patients with AHF. Fibroblast growth factor 21 (FGF21) is a member of the FGF family, and is expressed in heart, kidney and liver (6, 7). In addition, FGF21 is closely related to liver and renal function, and may be a potential biomarker of liver and renal damage (8, 9). FGF21 protects mouse liver against D-galactose-induced hepatocyte oxidative stress by activating PI3K/Akt pathway and enhancing Nrf2-mediated antioxidant capacity and apoptosis (10). FGF21 negatively regulates TGFβ-P53-Smad2/3 signaling, which mediates epithelial-mesenchymal transition by activating AKT, to alleviate renal fibrosis in mice (11). In patients with cardiovascular disease, circulating FGF21 concentration is closely related to prognosis. Shen et al. showed that higher baseline serum FGF21 is associated with CVD mortality in elderly patients during the 5-year follow-up (12). In addition, the circulating level of FGF21 is also related to the development and prognosis of hypertension and atherosclerosis (13). Experimental studies show that FGF21 activating MAPK signaling through binding to FGFR1c and its co-receptorβ- Klotho to prevent myocardial hypertrophy (14). Therefore, in AHF patients, the concentration of FGF21 in circulation may reflect the degree of multiple organ damage and may be an important biomarker for the prognosis of patients. To test this, we analyzed an AHF cohort to determine whether FGF21 can be useful to assess the prognosis of AHF during the follow-up.

## Materials and Methods

### Study Cohort

All investigations were in accordance with the principles of the declaration of Helsinki, and this research was approved by the ethics committee of the First Affiliated Hospital of Shantou University Medical College. All participants agreed to participate in the research by signing informed consent forms. In total, 402 AHF patients and 19 age- and sex-matched healthy controls were recruited from April 2019 to July 2021 in the First Affiliated Hospital of Shantou University Medical College. According to the 2016 ESC guidelines, the diagnosis of AHF was made according to the following three criteria: (1) new onset or deterioration of symptoms and signs, (2) NT-proBNP levels ≥300 pg/ml, and (3) echocardiographic evidence showed abnormal left ventricular activity in systolic or diastolic function (15). The 19 healthy controls were recruited from the Health Management Center in the First Affiliated Hospital of Shantou University Medical College. Healthy controls were excluded from coronary heart disease by resting electrocardiogram and echocardiography, as well as disease history.

### Follow-Up and Endpoints

After discharge from the hospital, patients were followed-up every 3 months by telephone interview. The primary endpoint of our study was all-cause death, and secondary endpoint was a composite endpoint of death and heart failure readmission. Treatments of all enrolled AHF patients were administered in accordance with the relevant treatment guidelines during the follow-up period, and additional information of the death and the course of death was obtained from the hospital admission records, death certificates and family members.

### Measurement of FGF21

Blood samples were obtained from patients within 24 h of hospital admission, and serum was collected by centrifugation at 4,000 rpm for 10 min and stored at −80°C. Serum FGF21 concentration was examined by ELISA kits (4A Biotech Co. Beijing, China) according to the instructions of the kits.

### Statistical Analysis

All statistical tests were conducted using SPSS statistical software (SPSS, Chicago, IL). The results are shown as the mean ± standard deviation (SD) for continuous variables with normal distribution, and as count (percentage) for categorical variables. A *t*-test or Mann-Whitney test was performed for comparisons between two groups. ANOVA and Kruskal-Wallis tests were used for comparison of multiple groups, and a *p* < 0.05 was considered to indicate significance. One-way ANOVA was used to test the trends of the FGF21 concentration in AHF patients. Univariate and multivariate Cox's proportional hazards model analyses were used to calculate the hazard ratios (HR) and 95% CI and independent prognostic factors. The Kaplan-Meier method was used to evaluate the distribution all-cause death and composite endpoint between the high FGF21 group and low FGF21 group, and a p-value of <0.05 was considered to indicate statistical significance. ROC was used to analysis the sensitivity and specificity of FGF21 and NT-proBNP for the all-cause death at 3 and 6 month after discharge.

## Results

**Figure 1:**
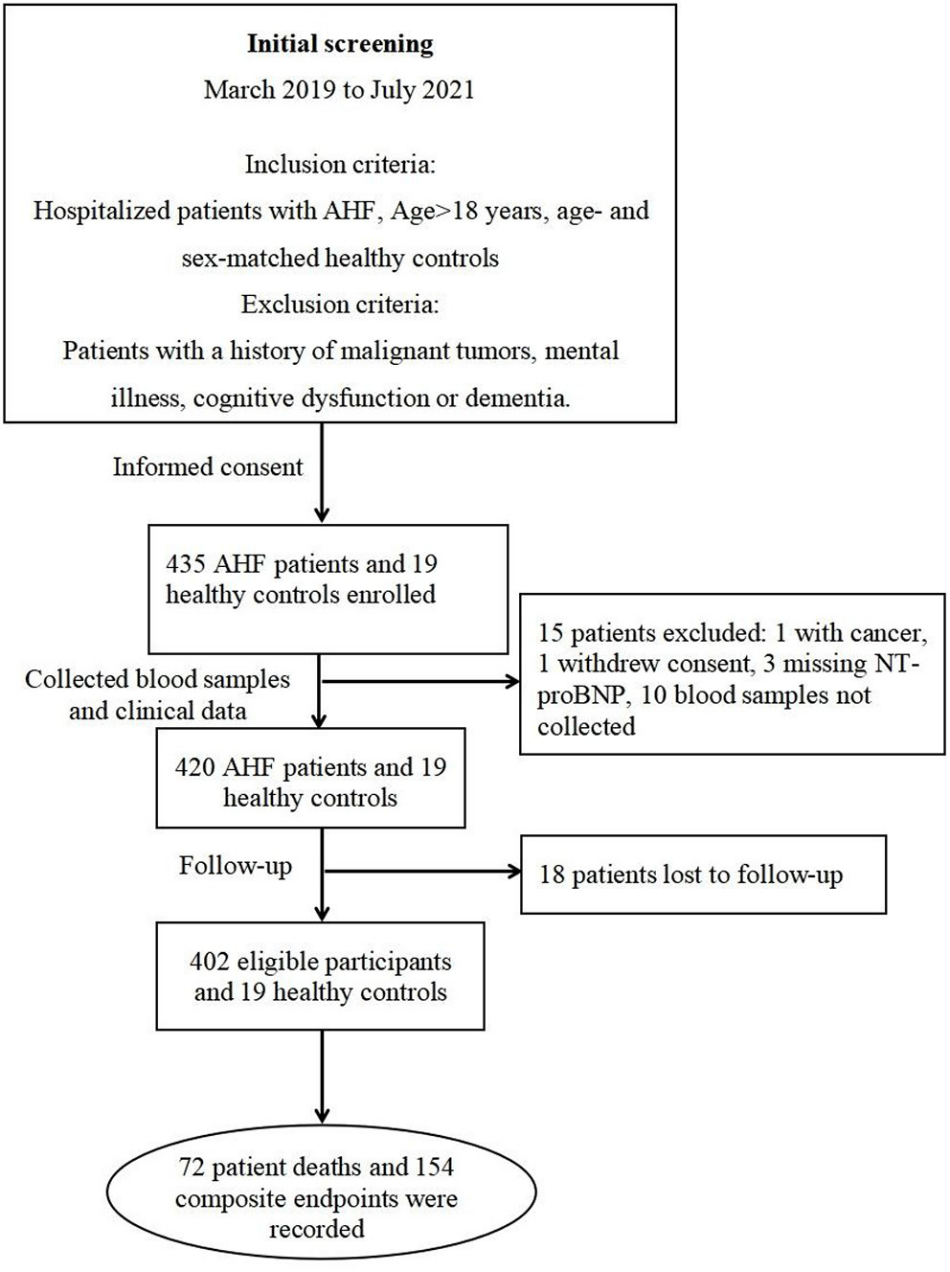
Flow chart of the study and outcome for the AHF cohort. AHF, acute heart failure.

During the study period, 435 AHF patients and 19 healthy controls were enrolled in our AHF cohort (Figure 1). Ultimately, 402 eligible patients and 19 healthy controls were included in the present study. The median follow-up was 190 days, during which all-cause death was 17.7% (72 patients), and the composite endpoint outcome was 38% (154 patients).

### Baseline Characteristics

The baseline clinical data of AHF patients are illustrated in Table 1. According to the median serum FGF21 level at admission, the AHF cohort was divided into high FGF21 (*n* = 201, FGF21 ≥ 262 pg/ml) and low FGF21 (*n* = 201, FGF21 <262 pg/ml) groups. The mean age was 70 ± 12 years, and more than half, 58% (234/402) were male. The high FGF21 group had higher BUN levels (10.9 (6.9–16.3) vs. 7.6 (5.7–10.9) mmol/L; *p* < 0.001), higher Cr (147 (106–206) vs. 108 (88–148) μmol/L; *p* < 0.001), higher TG (1.1 (0.9–1.5) vs. 0.9 (0.8–1.2) mmol/L; *p* < 0.001) and higher NT-proBNP (7.5 (3.1–11.1) vs. 3.8 (1.9–7.8) ng/ml; *p* < 0.001) compared with the low FGF21 group. Most importantly, the incidence of all-cause death and composite outcome events were higher in the high FGF21 group compared with the low FGF21 group (57 (28.3%) vs. 15 (7.4%); *p* < 0.001 and 101 (50%) vs. 53 (26.3%); *p* < 0.001) during the follow-up. These results indicate that high expression of FGF21 predicts poor prognosis in AHF patients.

**Table 1:** Baseline characteristics of the AHF cohort (*n* = 402).

	**AHF** **(*n =* 402)**	**FGF21-low group** **(*n =* 201)**	**FGF21-high** **(*n =* 201)**	**Health control** **(*n =* 19)**	***P*-value**
Age, yrs	70 ± 12	69 ± 12	71 ± 12	67 ± 7	0.476
Sex, male	234 (58%)	133 (66%)	101 (50%)	12 (63%)	0.002
**Comorbidity**					
Hypertension	266 (66%)	130 (64%)	136 (67%)	0	0.598
Diabetes mellitus	172 (43%)	82 (41%)	90 (45%)	0	0.481
Atrial fibrillation	127 (32%)	57 (28%)	70 (35%)	0	0198
Coronary heart disease	215 (53%)	107 (53%)	108 (54%)	0	1
Dilated cardiomyopathy	35 (9%)	18 (8.9%)	17 (8.5%)	0	1
**Measurements at admission**					
systolic blood pressure	139 (120–156)	139 (120–156)	139 (120–158)	130 (125–134)	0.632
diastolic blood pressure	84 (72–96)	84 (70–95)	84 (74–98)	76 (70–85)	0.445
LVEF, %	49 (39–61)	49 (40–61)	49 (39–61)	68 (65–72)	0.967
LVED	52 (46–58)	52 (46–58)	52 (47–58)	43 (40–46)	0.917
WBC, 10^9^/L	7.7 (6.2–9.9)	7.7 (6.2–10.2)	7.6 (6.2–9.9)	7.5 (6.9–8.6)	0.719
Hb, mg/L	121 (102–135)	126 (110–138)	117 (92–132)	132 (128–148)	0.001
BUN, mmol/L	8.6 (6.0–13.3)	7.6 (5.7–10.9)	10.9 (6.9–16.3)	5.0 (3.7–6.1)	0.001
Cr, μmol/L	123 (94–165)	108 (88–148)	147 (106–206)	75 (62–83)	0.001
ALB, g/L	34 ± 4	35 ± 4	33 ± 4	38 ± 12	0.238
ALT, IU/L	21 (14–41)	23 (15–39)	20 (12–45)	24 (17–32)	0.001
AST, IU/L	28 (20–44)	28 (21–39)	28 (19–48)	27 (22–37)	0.650
CKMB, IU/L	15.7 (12–21)	15 (12–21)	16 (12–21)	7 (5–8)	0.499
LDH, U/L	256 (213–332)	251 (217–326)	263 (208–342)	186 (145–227)	0.430
LDL-C, mmol/L	2.7 (2.2–3.2)	2.6 (2.2–3.1)	2.7 (2.1–3.3)	2.7 (2.2–3.7)	0.328
HDL-C, mmol/L	1.0 (0.8–1.2)	1.0 (0.9–1.2)	0.9 (0.7–1.1)	1.2 (1.0–1.4)	0.013
Chol, mmol/L	4.2 (3.4–5.1)	4.1 (3.5–4.9)	4.2 (3.4–5.3)	4.8 (3.7–5.4)	0.408
TG, mmol/L	1.0 (0.8–1.3)	0.9 (0.8–1.2)	1.1 (0.9–1.5)	1.3 (0.9–2.1)	0.001
NT-proBNP, ng/ml	5.3 (2.4–9.0)	3.8 (1.9–7.8)	7.5 (3.1–11.1)	0.4 (0.2-.05)	0.001
FGF21,pg/ml	262 (119–586)	/	/	87 (55–97)	0.001
**Medications at discharge**					
ACEIs/ARBs	231 (57.5%)	118 (58.7)	113 (56.2%)	NA	0.687
Beta-blockers	269 (66.9%)	141 (70.1%)	128 (63.7%)	NA	0.203
Loop diuretics	349 (86.8%)	171 (85.1%)	178 (88.6%)	NA	0.377
Statins	237 (59%)	119 (59.2%)	118 (58.7%)	NA	1
CCB	106 (26.4%)	50 (24.9%)	56 (27.9%)	NA	0.572
NYHA					0.001
II	63 (15.7%)	49 (24.4%)	14 (7%)	NA	
III	224 (55.7%)	103 (51.2%)	121 (54.0%)	NA	
IV	115 (28.6%)	49 (42.6%)	66 (32.8%)	NA	
**Outcome**					
All-cause death	72 (17.9%)	15 (7.4%)	57 (28.3%)	0	0.001
Composite endpoints	154 (38.3%)	53 (26.3%)	101 (50%)	0	0.001

*According to the median FGF21 level, the AHF cohort was divided into two groups: a low group (<262 pg/ml, n = 201) and a high group (≥262 pg/ml, n = 201). Continuous variables are presented as the mean ± SD or the median with the IQRs (25th, 75th percentiles). Categorical variables are presented as counts and percentages*.

*AHF, acute Heart Failure; ALB, albumin; ALT, alanine aminotransferase; ACEI/ARB, angiotensin-conberting enzyme enhibitors/angiotensin receptor blocker; BUN, blood urea nitrogen; Cr, creatinine; CKMB, creatinine kinase isoenzyme; HDL-C, high-density lipopritein cholesterol; Hb, hemoglobin; LVEF, left ventricular ejection fraction; LDH, lactate dehydrogenase; LDL-C, low-density lipoprotein cholesterol; NT-proBNP, N-terminal pro B-type natriuretic peptide; TG, triglyceride; WBC, white blood cells*.

### Effect of FGF21 and NT-ProBNP on Cardiac Function in Patients With Heart Failure

**Figure 2:**
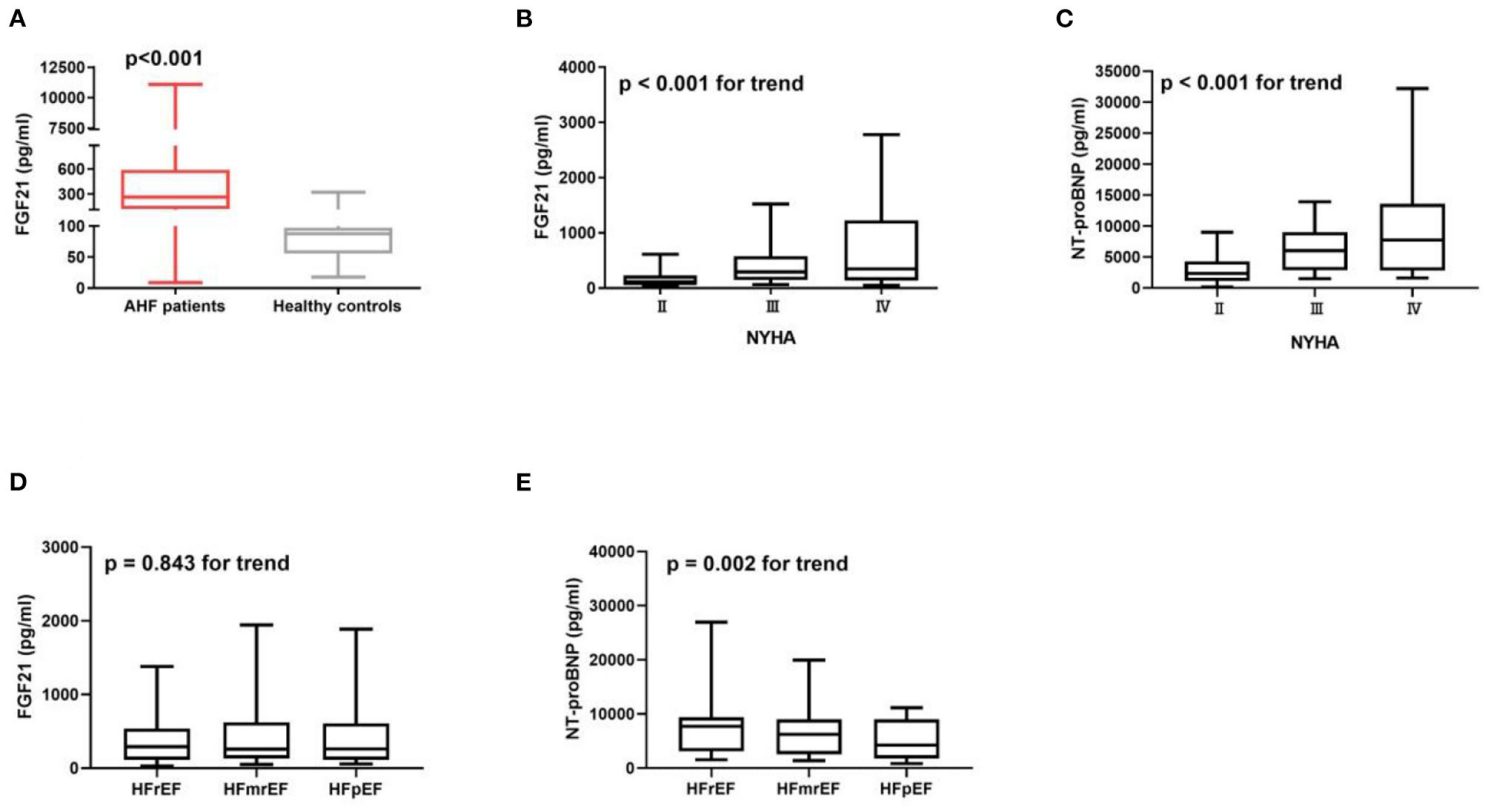
Serum FGF21 is higher in AHF patients (*n* = 402) compared to the healthy controls (*n* = 19) (*p* < 0.001) **(A)**. Concentration of FGF21 **(B)** and NT-proBNP **(C)** were increased with the increase in New York Heart Association (NYHA) functional class (*p* < 0.001 for trend). NT-proBNP gradually decreased from HFrEF, HFmrEF to HFpEF patients (*p* = 0.002 for trend) **(E)**, but not FGF21 (*p* = 0.843 for trend) **(D)**.

Serum FGF21 was higher in AHF patients compared with healthy controls (Figure 2A). FGF21 (Figure 2B) and NT-proBNP (Figure 2C) increased along with the increase in New York Heart Association (NYHA) functional class (*p* < 0.001 for trend). NT-proBNP gradually decreased from heart failure with reduced ejection fraction (HFrEF), heart failure with mid-range ejection fraction (HFmrEF) to heart failure with preserved ejection fraction (HFpEF) (*p* = 0.002 for trend) (Figure 2E), but FGF21 did not (*p* = 0.843 for trend) (Figure 2D), in HFrEF, HFmrEF and HFpEF patients.

### Relationship Between Serum FGF21 Levels and Clinical Parameters

**Figure 3:**
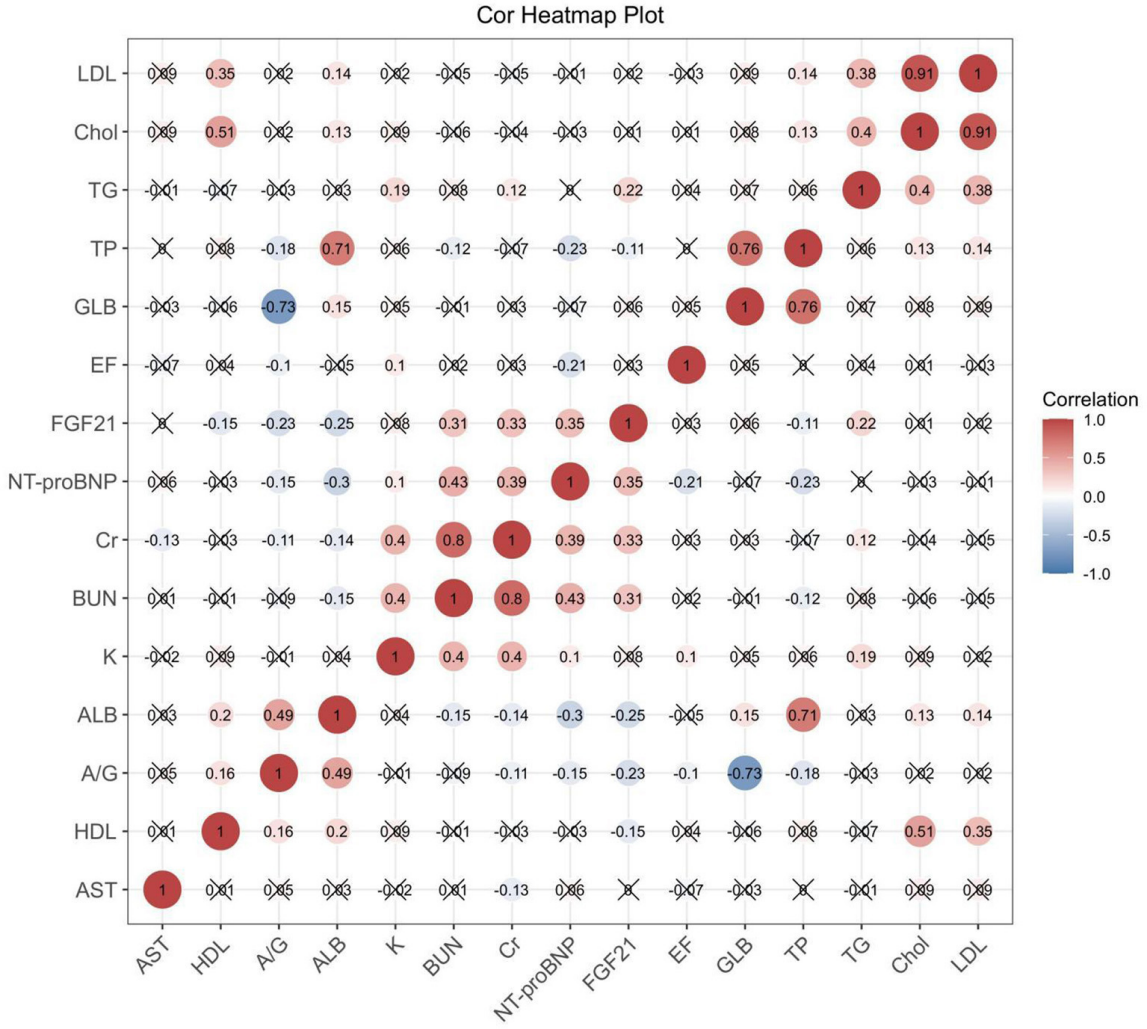
Correlation heat map shows the correlation between serum FGF21 and biochemical parameters, BNP, and EF values in AHF patients. Those with statistical significance (*p* < 0.05) are presented as dot plots. ALB, albumin; A/G, albumin/globulin; AST, aspartate aminotransferase; BUN, blood urea nitrogen; Cr, creatinine; Chol, cholesterol; GLB, globulin; HDL, high-density lipoprotein; LDL, low-density lipoprotein; NT-proBNP, N-terminal brain natriuretic peptide; TG, triglyceride; TP, total protein.

FGF21 was positively correlated with AST, BUN, Cr, NT-proBNP and TG, and negatively correlated with ALB and HDL (Figure 3). This suggests that FGF21 may be involved in multiple organ damage, such as heart, liver and kidney, and may be involved in the regulation of lipid metabolism.

### Relationship Between FGF21 and Clinical Prognosis

**Figure 4:**
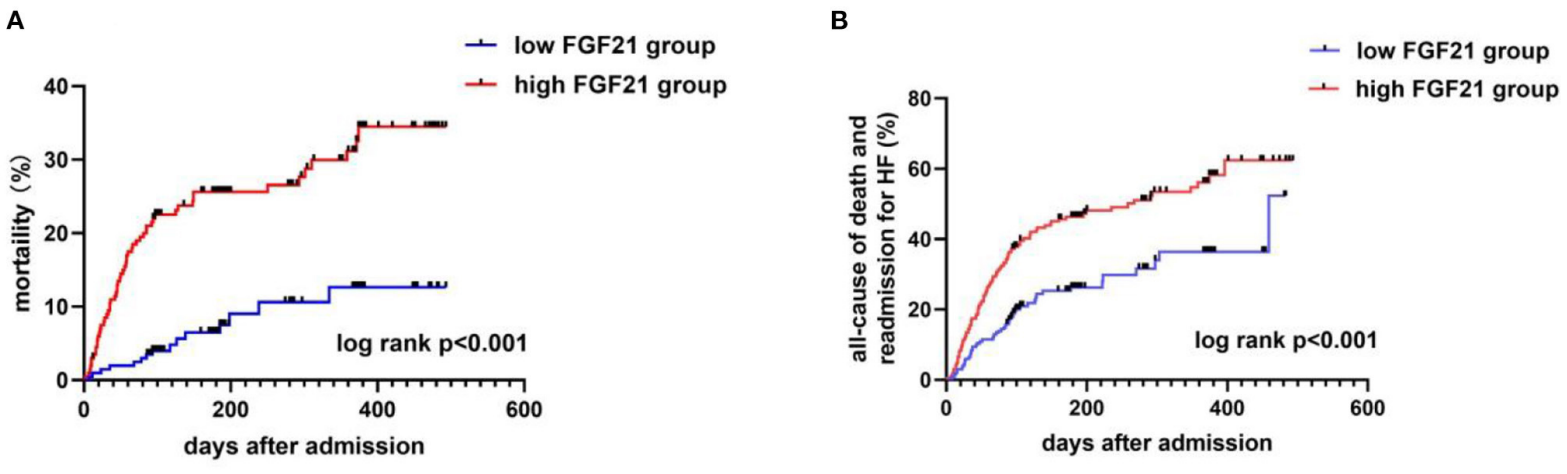
Kaplan-Meier curves for all-cause death **(A)** and composite endpoints **(B)** stratified by FGF21 median in AHF patients. Mortality **(A)** and composite endpoints **(B)** were significantly higher in the high FGF21 group than the low FGF21 group (*p* < 0.001).

**Figure 5:**
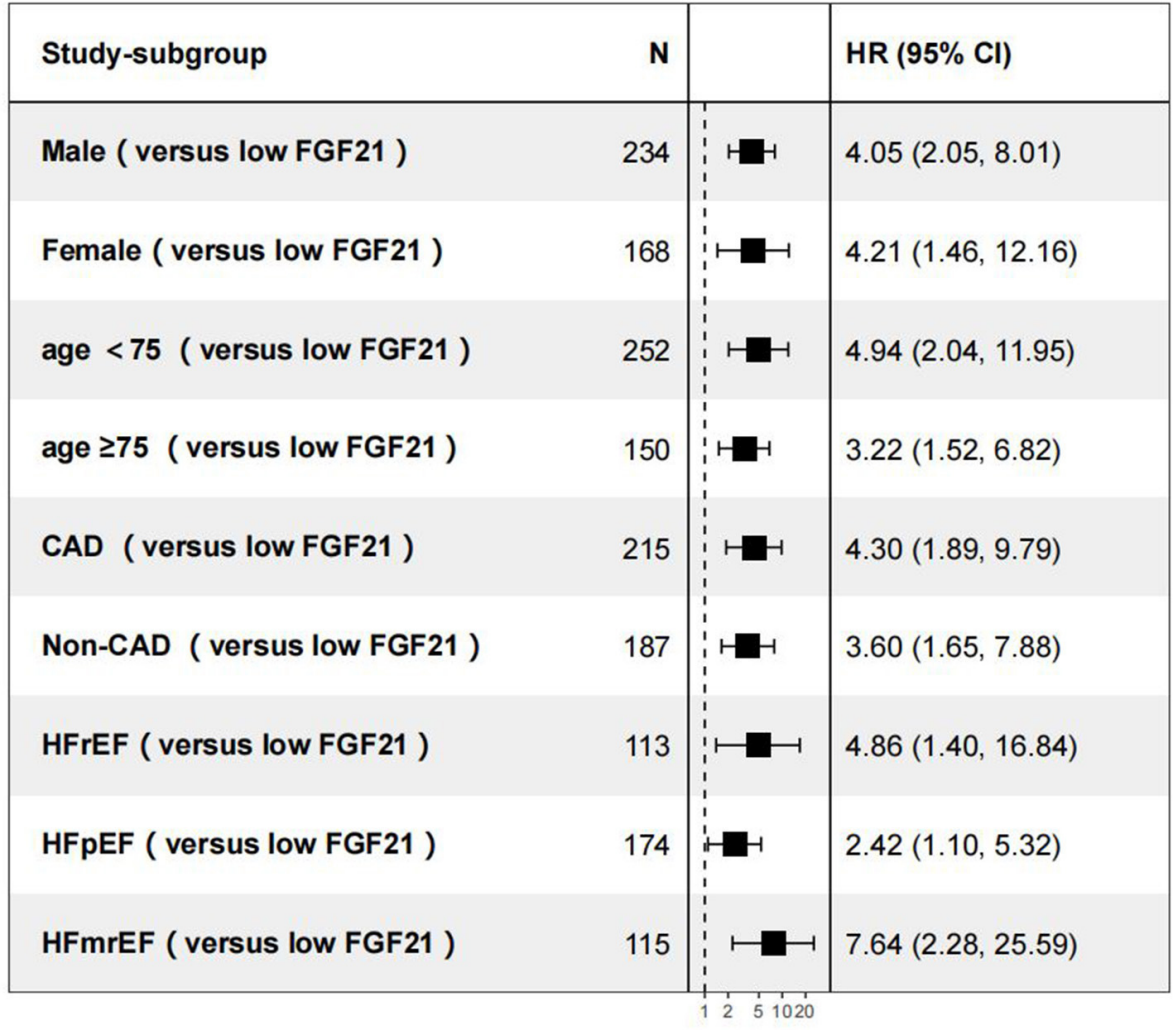
Subgroup analyses of all-cause death vs. the low FGF21 group. CAD, coronary artery disease; HFpEF, heart failure with preserved ejection fraction; HFrEF, heart failure with reduced ejection fraction.

During the days of follow-up, in our AHF cohort, 154 patients were in the composite endpoint group, among whom 72 died. Kaplan-Meier survival analysis demonstrated that AHF patients with higher serum FGF21 levels had significantly higher all-cause death risk compared with low serum FGF21 levels (*p* < 0.001) (Figure 4A), and also displayed the same trend for composite endpoints (*p* < 0.001) (Figure 4B). Univariate Cox regression indicated that high serum admission serum FGF21 levels were associated with increased risk of mortality during follow-up. Patients with high FGF21 levels had a higher risk of all-cause death, with an HR of 3.91 (95% CI, 2.21–6.92, *p* < 0.001)) vs. the low FGF21 group. After adjustment for age, sex, NT-proBNP, Cr, AST, Hb, BUN, NYHA class and EF, the high FGF21 group remained associated with increased risk for all-cause death (HR, 3.28, 95% CI 1.74–6.18, *p* < 0.001) (Table 2). FGF21 levels also were associated with the risk of all-cause death in subgroups (Figure 5). ROC analysis showed that FGF21 was better than NT-proBNP in predicting death at both 3 (AUC, 0.77 vs. 0.63, *p* < 0.001) and 6 months (AUC, 0.78 vs. 0.66) (Supplementary Figure 1).Thus, our study suggests that high serum FGF21 is a predictive biomarker for short-term prognosis in AHF patients.

**Table 2:** Risk factors for all-cause death according to the Cox proportional hazards regression model.

**Factors**		**Univariate analysis**		**Multivariable analysis**	
		**Hazard ratio** **(95% CI)**	***P***	**Hazard ratio** **(95% CI)**	***P***
Age		1.03 (1.00–1.05)	<0.001	1.02 (0.99–1.04)	0.14
Sex (vs. males)		0.83 (0.51–1.33)	0.44	0.54 (0.32–0.91)	0.02
NYHA(vs. class II)	Class III	2.03 (0.80–5.18)	0.136	1.21 (0.44–3.28)	0.711
	Class IV	3.27 (1.26–8.43)	0.014	1.64 (0.59–4.57)	0.348
FGF21 level (vs. low FGF21 group)		3.91 (2.21–6.92)	<0.001	3.28 (1.74–6.18)	<0.001
NT-proBNP ^*^		1.50 (1.27–1.76)	<0.001	1.16 (0.94–1.43)	0.169
FGF21^*^		1.50 (1.32–1.70)	<0.001		
Hb^*^		0.61 (0.49–0.74)	<0.001	0.80 (0.62–1.05)	0.11
BUN^*^		1.76 (1.49–2.08)	<0.001	1.74 (1.27–2.40)	<0.001
Cr^*^		1.35 (1.17–1.56)	<0.001		
A^ST*^		1.37 (1.18–1.60)	<0.001	2.09 (1.27–3.45)	0.004
HDL^*^		1.00 (0.79–1.28)	0.95	1.15 (0.89–1.50)	0.282
EF^*^		1.03 (0.82–1.30)	0.78	1.13 (0.87–1.46)	0.371

* *Per 1 SD. Cr, creatinine; AST, aspartate aminotransferase; BUN, blood urea nitrogen; EF, ejection fraction; HDL, high-density lipoprotein; NT-proBNP, N-terminal brain natriuretic peptide*.

## Discussion

In our study, we primarily investigated the association between in-hospital serum FGF21 levels and short-term outcomes in patients with AHF. Division of patients into high and low FGF21 groups, based on the median concentration of FGF21, showed short-term mortality and composite end point events were about 4- and 2-fold increased in the high FGF21 group compared with low FGF21 groups, even after adjustment for NT-proBNP, sex and age. These results suggest that FGF21 may be an important biomarker for the prognosis of AHF patients. The principal results of our study are 3-fold. First, serum levels of FGF21 measured in 24 h after admission are elevated in AHF patients compared with healthy controls. Second, the positive correlation, between serum FGF21 and NT-proBNP, Cr, BUN, TG, AST, indicates that the level of FGF21 is significantly correlated with severity of AHF. Third, FGF21 is associated with clinical outcome in AHF patients. Within 6 months after discharge, patients with higher levels of baseline serum FGF21 upon admission suffer from a higher risk of all-cause death and rehospitalization in AHF patients compared to patients with lower levels of serum FGF21, even after adjusting for NT-proBNP. These findings provide evidence that FGF21 may participate in the pathogenesis of AHF.

FGF21 is a hormone with the ability to regulate glucose and lipid metabolism through activation of the serum response factor/Ets-like protein-1 (16). It is mainly produced by the liver and also produced and secreted by heart (17, 18). The possible mechanism of FGF21 involvement in the regulation of prognosis in patients with AHF still needs to be explored at present.

Hospitalized patients with heart failure have the highest risk of death in 60 and 90 days after discharge. This early post-discharge period has been named the “vulnerable phase” and it is more important to predict the prognosis of heart failure patients during vulnerable phase (19). The role of FGF21 and BNP in the prognosis of chronic heart failure is similar. During 1-year follow-up, FGF21 and NT-proBNP have similar predictive effects in cardiovascular prognosis in HFpEF patients (20). FGF21 and NT-proBNP were equally effective in predicting all-cause death in systolic dysfunction patients during 5 years follow-up (12). In our study, FGF21 do better than NT-proBNP in prognosis of all-cause death in 3 and 6 month. The difference between our study and other studies is the time and population we follow-up, FGF21 is superior to NT-proBNP in short-term prognosis not in the long-term prognosis., which mean FGF21 may be more suitable for clinical using.

In previous study, using the optimal cutoff value of 321.5pg/mL, elevated baseline FGF21 predicted left ventricular systolic dysfunction independently (12). In another study, serum FGF21 concentration over 232 pg/ml independently predicted adverse atherosclerotic cardiovascular disease events in non-diabetic population (21). The cutoff value of FGF21which is similar to our study (262 pg/ml), However, more study should be done to explore the optimal cutoff values of FGF21 in predicting adverse outcome in AHF patients.

### FGF21 May Be Involved in the Progression of AHF by Regulating Lipid Metabolism

The main source of circulating FGF21 in AHF is still unknown, but the regulatory role of FGF21 in lipids has been demonstrated. FGF21 is regulated by PPARα to improve lipid levels in mice fed with a high fat, low carbohydrate ketogenic diet (22). In addition, serum FGF21 concentrations are increased in patients with non-alcoholic fatty liver disease, and are positively correlated with triglyceride levels, suggesting that FGF21 may be a potential biomarker of non-alcoholic fatty liver disease and be involved in the regulation of triglycerides (23). *In vitro*, many studies have shown that FGF21 regulates triglyceride metabolism through the following two mechanisms: first, FGF21 reduces the level of non-esterified fatty acids in blood; secondly, FGF21 accelerates the conversion of white adipose tissue to brown adipose tissue, thus accelerating triglyceride turnover and improving lipid metabolism (24, 25). FGF21 also improves cardiac function by improving lipid accumulation in diabetic mice (26). These studies confirm that FGF21 plays an important role in regulating lipid metabolism in disease states and participates in the development of disease. Our study also finds that serum FGF21 concentration is positively correlated with triglyceride and negatively correlated with high-density lipoprotein, suggesting that FGF21 may be involved in regulating the metabolism of triglyceride and high-density lipoprotein in patients with AHF. Potočnjak et al. showed that HDL cholesterol efflux capacity is an important risk factor for hospital death in patients with AHF. Another study showed that the concentration of small HDL particles is an important predictor of 3-month mortality in patients with AHF (27, 28). These results suggest that the function and concentration of lipoprotein may play an important role in the prognosis of AHF and could be regulated by FGF21. More experiments are needed to verify the effect of FGF21 on lipoprotein function.

As we all known, white adipose tissue (WAT) plays a crucial role in the development of cardiometabolic disorders, which could increased the prevalence of HF. FGF21 secret from WAT and browning WAT to keep balance (29). In another study, Higher plasma FGF21 levels were associated with higher pericardial fat volume at baseline which suggest that FGF21 may also through WAT to affect the development of HF (30).

### FGF21 May Regulate the Progression of AHF by Regulating Liver and Kidney Function

Due to the special interaction in heart, liver and kidney, heart failure patients are often accompanied with liver and kidney damage, which always indicates worse clinical prognosis (31–33). In our study, FGF21 was positively correlated with Cr (*r* = 0.33, *p* < 0.001) and BUN (*r* = 0.31, *p* < 0.001), which means that there are close relationship between FGF21 and renal function in AHF patients. Previous studies had shown that FGF21 play an important role in renal function, patients with renal impairment tend to have higher levels of FGF21 than normal individuals, and the higher the concentration of FGF21 in patients, the poorer the renal function (34–36). A recent study showed that mutations in FGF21-adjacent genes are associated with eGFR in Chinese diabetic patients (37), indicating the importance of FGF21 in renal function in some disease states. What role and how FGF21 plays in the kidneys is still no clear, but large studies had confirmed the protective effect of FGF21 in the kidneys. FGF21 prevents renal fibrosis by negatively regulating TGF-β/SMad2/3-mediated epithelial-to-mesenchymal transition in the diabetic mouse (11). FGF21 can prevent both hyperlipidemia and diabetes-induced renal damage, which was partially by reducing renal lipid accumulation and decreasing inflammation, oxidative stress and fibrosis. So we think that the increased concentration of FGF21 in AHF patients are associated with their renal function and FGF21 exerts a protective effect on the kidneys, which poorer kidney function means more circulating FGF21. Liver impairment is also common in patients with heart failure and is often associated with a poor cardiovascular outcome (5, 38). FGF21 can also be secreted by the liver, and recent studies have shown that FGF21 may be an early predictor of liver damage. In mouse models of alcoholic and non-alcoholic fatty liver, FGF21 can protect the liver by promoting autophagy as well as exert anti-inflammatory and antioxidant effects (39, 40). In D-galactose-induced oxidative stress of liver cells, FGF21 enhances Nrf2-mediated antioxidant capacity to protect the liver by activating the PI3K/Akt pathway (10). In patients with hyperthyroidism, serum FGF21 concentration is positively correlated with liver enzyme concentrations, suggesting that serum FGF21 concentration is related to liver function impairment in patients with hyperthyroidism (41). However, circulating FGF21 is not a biomarker of liver damage in patients with alcoholic cirrhosis (42). However, since the sample was small, more studies are needed to confirm this result. In conclusion, current studies support FGF21 as a biomarker of renal and liver damage and protect target organs from injury, which may be one of the reasons why FGF21 predicts the prognosis of patients in AHF patients.

### Mechanism of Negative Correlation Between FGF21 Level and Prognosis of AHF

The role of FGF21 in cardiovascular disease has also been discovered in recent years. Similar to our study, elevated FGF21 is associated with poor prognosis in cardiovascular disease. Coronary heart disease (CHD) patients have higher blood levels of FGF21, and patients with high concentrations of FGF21 tend to have poor lipid profiles (43). In a cohort of 670 subjects who underwent carotid intima-media thickness (IMT) measurements, elevated circulating FGF21 was associated with a higher risk of carotid atherosclerosis (44). In patients with diabetes, high serum levels of FGF21 increase the risk of adverse cardiovascular events during the 5 year follow-up period (45). In addition, serum FGF21 concentrations are elevated in patients with HFrEF patients, and elevated FGF21 may be associated with an inflammatory response *in vivo* and not with cardiac function (46). The elevated levels of FGF21 are closely associated with the increased of inflammation in the body, which may be the reason why elevated FGF21 always means poor prognosis in cardiovascular diseases. What is more, FGF21 protects myocardial cells from damage in many ways. Preconditioning mice with myocardial ischemia elevates FGF21 secretion by liver and adipose tissue, and reduces the activity of caspase 3 through the phosphorylation of PI3K to ultimately reduce myocardial cell death and apoptosis (47). In mice with diabetic cardiomyopathy, FGF21 protects the heart from inflammation and oxidative stress by activating Nrf2 to increase CD36 expression (48). In addition, FGF21 also protects cardiomyocytes by promoting increased levels of miR-145 and thus promoting autophagy in an ischemia-reperfusion mouse model (49). According to previous studies, elevated FGF21 represent the degree of damage and inflammation in the body which may be participate in the prognosis of AHF. There are two reasons to explain the elevated of FGF21. It is similar to insulin resistance, the patients suffers FGF21 resistance, so it means their body need to secrete more FGF21 for maintain the balance. Another reason is we secreted more FGF21 from liver and adipose tissue to protect our body from damage. However, the protective effect of FGF21 could not counteract the injury, so higher concentration of FGF21 in blood may represent larger damage in the body and always means poor prognosis in AHF patients.

There are also several shortcomings in this study. As a single-center study, the sample size is insufficient and the study lacks long-term follow-up data. Secondly, due to the close relationship between FGF21 and liver function, the patients in this study may have potential liver disease, but we lack additional information on liver pathology. Nevertheless, our study shows that higher serum FGF21 levels at baseline are associated with higher adverse cardiovascular events, possibly due to impairment of the FGF21 signaling pathway or compensatory protective responses to stress by our body.

## Data Availability Statement

The original contributions presented in the study are included in the article/Supplementary Material, further inquiries can be directed to the corresponding author.

## Ethics Statement

The studies involving human participants were reviewed and approved by Ethics Committee of the First Affiliated Hospital of Shantou University Medical College. The patients/participants provided their written informed consent to participate in this study.

## Author Contributions

GW: conception and methodology, project administration, and writing of the original draft. SW, JY, and MY: clinical data curation. JZ: writing, review, and editing. XT: writing, review, and editing. All authors contributed to the article and approved the submitted version.

## Conflict of Interest

The authors declare that the research was conducted in the absence of any commercial or financial relationships that could be construed as a potential conflict of interest.

## Publisher's Note

All claims expressed in this article are solely those of the authors and do not necessarily represent those of their affiliated organizations, or those of the publisher, the editors and the reviewers. Any product that may be evaluated in this article, or claim that may be made by its manufacturer, is not guaranteed or endorsed by the publisher.

## Supplementary Material

Supplementary Figure 1 Pairwise comparison of the ROC curve between FGF21 and NT-proBNP in predicting all-cause death at 3 **(A)** and 6 months **(B)** after discharge. FGF21 predicted all-cause deaths better than NT-proBNP for both 3 (*p* < 0.001) and 6 months (*p* < 0.001).
