# Supplementary figures and images for: Fibroblast Growth Factor 21 Predicts Short-Term Prognosis in Patients With Acute Heart Failure: A Prospective Cohort Study
Source: Front Cardiovasc Med. 2022 Mar 16;9:834967. doi: 10.3389/fcvm.2022.834967 (PMC8965840; doi:10.3389/fcvm.2022.834967)

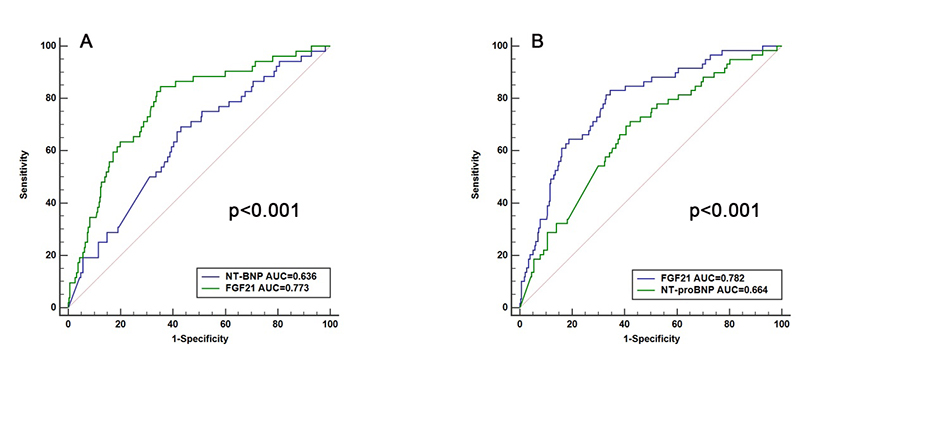

Supplement: Supplementary Figure 1 — Pairwise comparison of the ROC curve between FGF21 and NT-proBNP in predicting all-cause death at 3 (A) and 6 months (B) after discharge. FGF21 predicted all-cause deaths better than NT-proBNP for both 3 (p < 0.001) and 6 months (p < 0.001). [file Image_1.JPEG]
