# Supplementary material for: Fibroblast Growth Factor 21 Predicts Short-Term Prognosis in Patients With Acute Heart Failure: A Prospective Cohort Study
Source: Front Cardiovasc Med. 2022 Mar 16;9:834967. doi: 10.3389/fcvm.2022.834967 (PMC8965840; doi:10.3389/fcvm.2022.834967)
Supplement: Supplementary file 2 [file Table_1.DOCX]

gender/ age/ CHD/ DM/all-cuse death/FGF21/NT-proBNP

| 2.00  2.00  1.00  2.00  1.00  2.00  2.00  1.00  1.00  1.00  1.00  1.00  1.00  2.00  2.00  1.00  1.00  2.00  1.00  1.00  1.00  2.00  1.00  2.00  2.00  2.00  2.00  1.00  1.00  2.00  1.00  1.00  1.00  1.00  1.00  2.00  1.00  1.00  1.00  2.00  2.00  1.00  1.00  2.00  1.00  1.00  1.00  1.00  2.00  1.00  2.00  1.00  1.00  2.00  1.00  1.00  2.00  2.00  2.00  1.00  1.00  1.00  2.00  1.00  1.00  1.00  1.00  1.00  2.00  1.00  1.00  1.00  1.00  2.00  2.00  1.00  1.00  1.00  1.00  1.00  2.00  1.00  1.00  1.00  2.00  2.00  2.00  1.00  1.00  2.00  2.00  1.00  2.00  2.00  1.00  1.00  1.00  2.00  2.00  1.00  2.00  1.00  2.00  1.00  2.00  1.00  2.00  1.00  2.00  1.00  2.00  2.00  2.00  1.00  2.00  2.00  1.00  1.00  1.00  1.00  1.00  1.00  1.00  1.00  1.00  2.00  2.00  1.00  1.00  2.00  2.00  2.00  1.00  2.00  2.00  1.00  2.00  2.00  2.00  1.00  1.00  2.00  2.00  1.00  1.00  2.00  1.00  2.00  1.00  2.00  1.00  2.00  1.00  1.00  1.00  2.00  1.00  2.00  2.00  2.00  2.00  1.00  2.00  2.00  1.00  2.00  1.00  1.00  2.00  2.00  1.00  1.00  1.00  2.00  2.00  2.00  1.00  1.00  2.00  2.00  2.00  1.00  1.00  1.00  2.00  2.00  2.00  1.00  1.00  1.00  1.00  1.00  1.00  2.00  1.00  2.00  1.00  1.00  1.00  1.00  1.00  1.00  1.00  1.00  2.00  2.00  1.00  1.00  2.00  1.00  2.00  1.00  2.00  2.00  1.00  2.00  1.00  2.00  1.00  2.00  2.00  1.00  2.00  1.00  2.00  2.00  1.00  1.00  2.00  2.00  2.00  1.00  1.00  1.00  1.00  1.00  2.00  2.00  1.00  2.00  1.00  1.00  1.00  2.00  1.00  1.00  2.00  1.00  1.00  1.00  1.00  1.00  2.00  2.00  1.00  1.00  1.00  1.00  1.00  2.00  1.00  2.00  1.00  2.00  1.00  1.00  1.00  1.00  1.00  2.00  2.00  1.00  2.00  2.00  1.00  1.00  2.00  1.00  1.00  2.00  2.00  1.00  1.00  2.00  2.00  1.00  2.00  1.00  2.00  1.00  1.00  2.00  2.00  1.00  1.00  2.00  1.00  1.00  1.00  2.00  2.00  2.00  2.00  1.00  2.00  2.00  1.00  1.00  2.00  1.00  2.00  1.00  1.00  1.00  1.00  2.00  1.00  2.00  1.00  2.00  1.00  2.00  2.00  1.00  2.00  2.00  2.00  1.00  2.00  1.00  1.00  1.00  1.00  1.00  1.00  1.00  2.00  2.00  2.00  2.00  1.00  1.00  1.00  1.00  2.00  1.00  1.00  2.00  1.00  1.00  1.00  1.00  1.00  2.00  2.00  1.00  2.00  1.00  1.00  2.00  1.00  2.00  1.00  2.00  1.00  1.00  1.00  1.00  1.00  1.00  1.00  1.00  2.00  1.00  2.00  2.00  1.00  2.00  2.00  1.00  1.00  1.00  2.00  2.00  1.00  2.00  2.00  1.00  1.00  2.00  1.00  1.00  2.00  1.00  1.00  2.00  1.00  1.00  1.00  2.00  2.00  1.00 | 76  90  81  70  55  62  67  70  82  69  56  44  74  69  54  33  60  62  70  75  89  87  44  81  63  73  87  68  71  64  68  82  86  71  85  61  84  77  48  82  70  60  63  56  71  67  72  66  83  70  61  63  66  66  56  70  86  78  90  62  58  76  80  76  48  71  83  62  68  74  58  84  66  80  68  90  59  80  89  53  55  72  62  79  72  68  64  55  58  75  77  67  87  80  78  67  70  79  52  70  75  33  67  81  64  74  62  63  84  77  76  71  79  70  68  70  57  61  75  75  68  77  20  67  70  80  70  85  84  71  67  71  79  65  74  61  69  65  65  73  66  51  86  60  62  65  62  61  72  60  49  85  63  73  58  61  84  83  70  77  72  51  66  74  78  85  100  25  89  85  80  72  68  52  77  88  82  84  64  70  79  90  83  76  86  86  74  56  70  80  67  75  81  62  50  81  37  87  65  70  73  68  69  76  84  90  82  74  69  58  91  85  63  56  79  81  71  61  54  74  87  69  68  59  76  86  66  63  50  71  68  60  60  73  72  82  73  68  77  76  57  68  53  71  66  71  73  72  73  69  74  86  61  64  69  88  75  68  89  75  68  85  87  86  69  49  79  82  85  74  67  34  79  58  87  71  48  71  74  88  67  66  68  74  87  77  66  71  73  63  68  74  85  81  81  41  76  87  77  73  85  68  85  75  81  80  81  65  67  46  78  87  71  71  72  89  71  58  83  75  80  82  75  54  71  66  66  87  63  57  77  75  42  70  88  66  73  71  63  61  58  88  71  50  85  83  87  66  56  61  91  56  70  55  76  61  89  81  75  68  61  75  43  63  70  77  55  93  89  66  79  74  63  38  68  63  59  68  87  89  65  56  76  73  49  66  75  38  63  69  63  46  72  73  79  86  91  55  72  66  84  53 | \| 1 \| \| --- \| \| 1 \| \| 0 \| \| 0 \| \| 0 \| \| 0 \| \| 0 \| \| 1 \| \| 1 \| \| 0 \| \| 1 \| \| 0 \| \| 0 \| \| 1 \| \| 0 \| \| 0 \| \| 1 \| \| 0 \| \| 0 \| \| 1 \| \| 1 \| \| 1 \| \| 0 \| \| 0 \| \| 1 \| \| 1 \| \| 0 \| \| 1 \| \| 1 \| \| 0 \| \| 1 \| \| 1 \| \| 1 \| \| 1 \| \| 1 \| \| 1 \| \| 0 \| \| 1 \| \| 1 \| \| 0 \| \| 0 \| \| 1 \| \| 1 \| \| 0 \| \| 1 \| \| 0 \| \| 1 \| \| 1 \| \| 0 \| \| 1 \| \| 1 \| \| 0 \| \| 1 \| \| 1 \| \| 1 \| \| 1 \| \| 1 \| \| 0 \| \| 0 \| \| 0 \| \| 1 \| \| 0 \| \| 0 \| \| 1 \| \| 1 \| \| 0 \| \| 0 \| \| 1 \| \| 1 \| \| 1 \| \| 1 \| \| 1 \| \| 0 \| \| 0 \| \| 1 \| \| 1 \| \| 1 \| \| 1 \| \| 0 \| \| 1 \| \| 1 \| \| 1 \| \| 1 \| \| 1 \| \| 1 \| \| 0 \| \| 1 \| \| 0 \| \| 1 \| \| 1 \| \| 0 \| \| 1 \| \| 0 \| \| 0 \| \| 1 \| \| 0 \| \| 1 \| \| 1 \| \| 0 \| \| 0 \| \| 0 \| \| 0 \| \| 0 \| \| 0 \| \| 1 \| \| 1 \| \| 1 \| \| 0 \| \| 0 \| \| 1 \| \| 1 \| \| 0 \| \| 1 \| \| 1 \| \| 0 \| \| 1 \| \| 0 \| \| 0 \| \| 0 \| \| 1 \| \| 1 \| \| 0 \| \| 1 \| \| 1 \| \| 1 \| \| 0 \| \| 1 \| \| 1 \| \| 1 \| \| 1 \| \| 1 \| \| 0 \| \| 1 \| \| 0 \| \| 1 \| \| 0 \| \| 1 \| \| 1 \| \| 0 \| \| 0 \| \| 1 \| \| 0 \| \| 1 \| \| 1 \| \| 1 \| \| 0 \| \| 0 \| \| 0 \| \| 1 \| \| 1 \| \| 1 \| \| 1 \| \| 0 \| \| 0 \| \| 1 \| \| 0 \| \| 0 \| \| 1 \| \| 0 \| \| 1 \| \| 0 \| \| 1 \| \| 1 \| \| 0 \| \| 1 \| \| 0 \| \| 0 \| \| 0 \| \| 1 \| \| 0 \| \| 0 \| \| 1 \| \| 1 \| \| 0 \| \| 0 \| \| 1 \| \| 1 \| \| 1 \| \| 1 \| \| 0 \| \| 1 \| \| 1 \| \| 0 \| \| 0 \| \| 0 \| \| 1 \| \| 0 \| \| 1 \| \| 0 \| \| 0 \| \| 0 \| \| 0 \| \| 1 \| \| 1 \| \| 0 \| \| 0 \| \| 0 \| \| 1 \| \| 1 \| \| 0 \| \| 0 \| \| 0 \| \| 0 \| \| 1 \| \| 1 \| \| 0 \| \| 0 \| \| 1 \| \| 1 \| \| 0 \| \| 0 \| \| 1 \| \| 0 \| \| 1 \| \| 0 \| \| 1 \| \| 1 \| \| 0 \| \| 0 \| \| 1 \| \| 0 \| \| 1 \| \| 0 \| \| 0 \| \| 0 \| \| 0 \| \| 1 \| \| 1 \| \| 1 \| \| 0 \| \| 1 \| \| 0 \| \| 1 \| \| 1 \| \| 0 \| \| 1 \| \| 1 \| \| 1 \| \| 0 \| \| 1 \| \| 1 \| \| 1 \| \| 0 \| \| 0 \| \| 0 \| \| 1 \| \| 0 \| \| 1 \| \| 1 \| \| 0 \| \| 1 \| \| 1 \| \| 1 \| \| 0 \| \| 0 \| \| 0 \| \| 1 \| \| 1 \| \| 1 \| \| 1 \| \| 1 \| \| 1 \| \| 0 \| \| 1 \| \| 0 \| \| 0 \| \| 0 \| \| 0 \| \| 1 \| \| 1 \| \| 0 \| \| 0 \| \| 0 \| \| 1 \| \| 1 \| \| 0 \| \| 0 \| \| 1 \| \| 1 \| \| 0 \| \| 1 \| \| 1 \| \| 1 \| \| 0 \| \| 1 \| \| 1 \| \| 1 \| \| 0 \| \| 1 \| \| 1 \| \| 0 \| \| 0 \| \| 0 \| \| 1 \| \| 0 \| \| 0 \| \| 1 \| \| 0 \| \| 0 \| \| 1 \| \| 0 \| \| 0 \| \| 0 \| \| 1 \| \| 1 \| \| 0 \| \| 0 \| \| 1 \| \| 0 \| \| 0 \| \| 1 \| \| 0 \| \| 1 \| \| 0 \| \| 1 \| \| 1 \| \| 0 \| \| 0 \| \| 1 \| \| 1 \| \| 1 \| \| 1 \| \| 1 \| \| 1 \| \| 0 \| \| 0 \| \| 1 \| \| 1 \| \| 1 \| \| 1 \| \| 1 \| \| 0 \| \| 1 \| \| 1 \| \| 1 \| \| 1 \| \| 0 \| \| 0 \| \| 1 \| \| 0 \| \| 1 \| \| 1 \| \| 1 \| \| 0 \| \| 1 \| \| 0 \| \| 1 \| \| 1 \| \| 1 \| \| 1 \| \| 1 \| \| 1 \| \| 1 \| \| 0 \| \| 1 \| \| 0 \| \| 0 \| \| 0 \| \| 1 \| \| 0 \| \| 1 \| \| 1 \| \| 0 \| \| 1 \| \| 1 \| \| 1 \| \| 1 \| \| 1 \| \| 0 \| \| 0 \| \| 0 \| \| 1 \| \| 1 \| \| 0 \| \| 0 \| \| 1 \| \| 1 \| \| 0 \| \| 0 \| \| 0 \| \| 0 \| \| 0 \| \| 0 \| \| 0 \| \| 0 \| \| 0 \| \| 1 \| \| 0 \| \| 0 \| \| 0 \| \| 0 \| \| 0 \| \| 0 \| \| 0 \| \| 1 \| \| 0 \| \| 1 \| \| 0 \| \| 1 \| \| 1 \| \| 1 \| \| 0 \| | \| 1 \| \| --- \| \| 1 \| \| 1 \| \| 0 \| \| 0 \| \| 0 \| \| 1 \| \| 0 \| \| 0 \| \| 1 \| \| 1 \| \| 0 \| \| 1 \| \| 1 \| \| 0 \| \| 0 \| \| 1 \| \| 0 \| \| 0 \| \| 0 \| \| 0 \| \| 0 \| \| 0 \| \| 1 \| \| 1 \| \| 1 \| \| 0 \| \| 0 \| \| 1 \| \| 0 \| \| 1 \| \| 0 \| \| 0 \| \| 0 \| \| 1 \| \| 0 \| \| 1 \| \| 1 \| \| 0 \| \| 1 \| \| 1 \| \| 1 \| \| 1 \| \| 1 \| \| 1 \| \| 0 \| \| 1 \| \| 0 \| \| 0 \| \| 1 \| \| 1 \| \| 0 \| \| 1 \| \| 0 \| \| 0 \| \| 1 \| \| 0 \| \| 0 \| \| 0 \| \| 0 \| \| 0 \| \| 0 \| \| 0 \| \| 0 \| \| 0 \| \| 0 \| \| 0 \| \| 1 \| \| 1 \| \| 0 \| \| 0 \| \| 0 \| \| 0 \| \| 0 \| \| 1 \| \| 1 \| \| 1 \| \| 0 \| \| 0 \| \| 1 \| \| 1 \| \| 0 \| \| 0 \| \| 0 \| \| 0 \| \| 0 \| \| 1 \| \| 0 \| \| 1 \| \| 1 \| \| 1 \| \| 0 \| \| 0 \| \| 1 \| \| 0 \| \| 1 \| \| 1 \| \| 0 \| \| 0 \| \| 0 \| \| 0 \| \| 1 \| \| 1 \| \| 0 \| \| 1 \| \| 0 \| \| 1 \| \| 0 \| \| 0 \| \| 1 \| \| 1 \| \| 0 \| \| 0 \| \| 1 \| \| 1 \| \| 0 \| \| 1 \| \| 1 \| \| 0 \| \| 1 \| \| 1 \| \| 0 \| \| 0 \| \| 0 \| \| 1 \| \| 0 \| \| 1 \| \| 1 \| \| 0 \| \| 1 \| \| 1 \| \| 0 \| \| 0 \| \| 0 \| \| 0 \| \| 0 \| \| 1 \| \| 1 \| \| 0 \| \| 1 \| \| 0 \| \| 0 \| \| 1 \| \| 1 \| \| 0 \| \| 1 \| \| 1 \| \| 0 \| \| 0 \| \| 1 \| \| 0 \| \| 1 \| \| 1 \| \| 0 \| \| 1 \| \| 0 \| \| 0 \| \| 1 \| \| 0 \| \| 1 \| \| 1 \| \| 0 \| \| 1 \| \| 1 \| \| 1 \| \| 1 \| \| 0 \| \| 0 \| \| 1 \| \| 0 \| \| 1 \| \| 0 \| \| 1 \| \| 1 \| \| 1 \| \| 0 \| \| 0 \| \| 1 \| \| 1 \| \| 0 \| \| 1 \| \| 0 \| \| 1 \| \| 1 \| \| 1 \| \| 1 \| \| 1 \| \| 1 \| \| 0 \| \| 1 \| \| 1 \| \| 0 \| \| 1 \| \| 1 \| \| 1 \| \| 0 \| \| 0 \| \| 0 \| \| 1 \| \| 0 \| \| 1 \| \| 0 \| \| 0 \| \| 1 \| \| 1 \| \| 0 \| \| 0 \| \| 1 \| \| 1 \| \| 0 \| \| 0 \| \| 0 \| \| 1 \| \| 0 \| \| 0 \| \| 0 \| \| 0 \| \| 1 \| \| 0 \| \| 1 \| \| 0 \| \| 1 \| \| 0 \| \| 0 \| \| 0 \| \| 0 \| \| 0 \| \| 0 \| \| 1 \| \| 1 \| \| 0 \| \| 1 \| \| 1 \| \| 0 \| \| 0 \| \| 1 \| \| 1 \| \| 0 \| \| 1 \| \| 1 \| \| 1 \| \| 1 \| \| 0 \| \| 1 \| \| 0 \| \| 1 \| \| 0 \| \| 1 \| \| 1 \| \| 1 \| \| 0 \| \| 0 \| \| 1 \| \| 1 \| \| 1 \| \| 0 \| \| 0 \| \| 1 \| \| 1 \| \| 1 \| \| 1 \| \| 0 \| \| 0 \| \| 1 \| \| 0 \| \| 0 \| \| 1 \| \| 0 \| \| 1 \| \| 1 \| \| 0 \| \| 0 \| \| 0 \| \| 0 \| \| 1 \| \| 0 \| \| 0 \| \| 1 \| \| 1 \| \| 1 \| \| 1 \| \| 1 \| \| 1 \| \| 0 \| \| 0 \| \| 0 \| \| 0 \| \| 0 \| \| 1 \| \| 1 \| \| 1 \| \| 0 \| \| 0 \| \| 0 \| \| 0 \| \| 0 \| \| 0 \| \| 1 \| \| 0 \| \| 1 \| \| 0 \| \| 0 \| \| 0 \| \| 1 \| \| 1 \| \| 1 \| \| 0 \| \| 1 \| \| 1 \| \| 0 \| \| 0 \| \| 0 \| \| 0 \| \| 0 \| \| 0 \| \| 1 \| \| 0 \| \| 1 \| \| 0 \| \| 0 \| \| 0 \| \| 1 \| \| 0 \| \| 0 \| \| 0 \| \| 0 \| \| 0 \| \| 1 \| \| 0 \| \| 0 \| \| 0 \| \| 0 \| \| 1 \| \| 0 \| \| 0 \| \| 0 \| \| 1 \| \| 1 \| \| 0 \| \| 0 \| \| 1 \| \| 0 \| \| 0 \| \| 0 \| \| 0 \| \| 0 \| \| 1 \| \| 0 \| \| 0 \| \| 1 \| \| 0 \| \| 0 \| \| 0 \| \| 0 \| \| 1 \| \| 0 \| \| 0 \| \| 0 \| \| 1 \| \| 0 \| \| 0 \| \| 1 \| \| 0 \| \| 1 \| \| 1 \| \| 0 \| \| 0 \| \| 0 \| \| 0 \| \| 0 \| \| 1 \| \| 0 \| \| 1 \| \| 0 \| \| 0 \| \| 1 \| \| 0 \| \| 0 \| \| 0 \| \| 0 \| \| 1 \| \| 0 \| \| 0 \| \| 0 \| \| 0 \| \| 1 \| \| 1 \| \| 0 \| \| 0 \| \| 0 \| \| 0 \| \| 0 \| \| 0 \| \| 0 \| \| 1 \| \| 0 \| \| 0 \| \| 0 \| \| 1 \| \| 0 \| \| 0 \| \| 0 \| | \| 1 \| \| --- \| \| 0 \| \| 0 \| \| 1 \| \| 0 \| \| 0 \| \| 0 \| \| 0 \| \| 1 \| \| 1 \| \| 0 \| \| 0 \| \| 1 \| \| 0 \| \| 0 \| \| 0 \| \| 0 \| \| 0 \| \| 0 \| \| 0 \| \| 0 \| \| 0 \| \| 0 \| \| 0 \| \| 0 \| \| 0 \| \| 0 \| \| 0 \| \| 0 \| \| 0 \| \| 0 \| \| 1 \| \| 1 \| \| 0 \| \| 1 \| \| 0 \| \| 1 \| \| 1 \| \| 1 \| \| 0 \| \| 0 \| \| 0 \| \| 1 \| \| 0 \| \| 0 \| \| 0 \| \| 0 \| \| 0 \| \| 0 \| \| 0 \| \| 1 \| \| 0 \| \| 1 \| \| 0 \| \| 0 \| \| 1 \| \| 1 \| \| 0 \| \| 0 \| \| 0 \| \| 0 \| \| 1 \| \| 0 \| \| 0 \| \| 0 \| \| 0 \| \| 0 \| \| 0 \| \| 0 \| \| 1 \| \| 0 \| \| 0 \| \| 0 \| \| 0 \| \| 0 \| \| 0 \| \| 0 \| \| 0 \| \| 0 \| \| 0 \| \| 0 \| \| 0 \| \| 0 \| \| 0 \| \| 0 \| \| 0 \| \| 0 \| \| 0 \| \| 0 \| \| 0 \| \| 0 \| \| 0 \| \| 0 \| \| 1 \| \| 0 \| \| 1 \| \| 0 \| \| 0 \| \| 1 \| \| 1 \| \| 0 \| \| 0 \| \| 0 \| \| 0 \| \| 0 \| \| 0 \| \| 0 \| \| 0 \| \| 0 \| \| 0 \| \| 0 \| \| 0 \| \| 0 \| \| 0 \| \| 0 \| \| 0 \| \| 1 \| \| 0 \| \| 1 \| \| 0 \| \| 0 \| \| 0 \| \| 1 \| \| 1 \| \| 0 \| \| 0 \| \| 0 \| \| 0 \| \| 1 \| \| 1 \| \| 1 \| \| 0 \| \| 0 \| \| 0 \| \| 0 \| \| 0 \| \| 0 \| \| 0 \| \| 0 \| \| 0 \| \| 0 \| \| 0 \| \| 0 \| \| 0 \| \| 0 \| \| 1 \| \| 0 \| \| 1 \| \| 0 \| \| 0 \| \| 0 \| \| 0 \| \| 0 \| \| 0 \| \| 0 \| \| 0 \| \| 0 \| \| 1 \| \| 1 \| \| 0 \| \| 0 \| \| 0 \| \| 0 \| \| 0 \| \| 0 \| \| 0 \| \| 0 \| \| 0 \| \| 1 \| \| 0 \| \| 0 \| \| 0 \| \| 1 \| \| 0 \| \| 0 \| \| 0 \| \| 1 \| \| 0 \| \| 1 \| \| 0 \| \| 0 \| \| 1 \| \| 0 \| \| 0 \| \| 0 \| \| 0 \| \| 0 \| \| 0 \| \| 0 \| \| 0 \| \| 0 \| \| 0 \| \| 1 \| \| 0 \| \| 0 \| \| 0 \| \| 0 \| \| 0 \| \| 0 \| \| 1 \| \| 0 \| \| 0 \| \| 0 \| \| 1 \| \| 1 \| \| 0 \| \| 0 \| \| 0 \| \| 0 \| \| 0 \| \| 0 \| \| 0 \| \| 0 \| \| 0 \| \| 0 \| \| 0 \| \| 0 \| \| 0 \| \| 0 \| \| 0 \| \| 0 \| \| 0 \| \| 0 \| \| 0 \| \| 0 \| \| 0 \| \| 0 \| \| 0 \| \| 0 \| \| 0 \| \| 0 \| \| 1 \| \| 0 \| \| 0 \| \| 0 \| \| 0 \| \| 0 \| \| 0 \| \| 1 \| \| 0 \| \| 0 \| \| 0 \| \| 1 \| \| 0 \| \| 0 \| \| 0 \| \| 0 \| \| 0 \| \| 0 \| \| 0 \| \| 1 \| \| 0 \| \| 1 \| \| 0 \| \| 0 \| \| 1 \| \| 1 \| \| 0 \| \| 0 \| \| 0 \| \| 0 \| \| 0 \| \| 0 \| \| 0 \| \| 0 \| \| 0 \| \| 0 \| \| 0 \| \| 0 \| \| 1 \| \| 0 \| \| 0 \| \| 0 \| \| 0 \| \| 1 \| \| 1 \| \| 0 \| \| 0 \| \| 0 \| \| 1 \| \| 0 \| \| 0 \| \| 0 \| \| 0 \| \| 1 \| \| 1 \| \| 0 \| \| 0 \| \| 0 \| \| 0 \| \| 0 \| \| 0 \| \| 0 \| \| 0 \| \| 0 \| \| 0 \| \| 0 \| \| 0 \| \| 1 \| \| 0 \| \| 1 \| \| 0 \| \| 1 \| \| 0 \| \| 0 \| \| 0 \| \| 0 \| \| 0 \| \| 0 \| \| 0 \| \| 0 \| \| 1 \| \| 0 \| \| 1 \| \| 0 \| \| 1 \| \| 0 \| \| 1 \| \| 0 \| \| 0 \| \| 0 \| \| 0 \| \| 0 \| \| 0 \| \| 0 \| \| 1 \| \| 0 \| \| 0 \| \| 0 \| \| 0 \| \| 0 \| \| 1 \| \| 0 \| \| 0 \| \| 0 \| \| 0 \| \| 0 \| \| 0 \| \| 0 \| \| 1 \| \| 0 \| \| 0 \| \| 0 \| \| 0 \| \| 0 \| \| 0 \| \| 0 \| \| 0 \| \| 0 \| \| 0 \| \| 1 \| \| 0 \| \| 0 \| \| 0 \| \| 0 \| \| 0 \| \| 0 \| \| 0 \| \| 0 \| \| 0 \| \| 0 \| \| 0 \| \| 0 \| \| 0 \| \| 0 \| \| 0 \| \| 0 \| \| 0 \| \| 1 \| \| 0 \| \| 0 \| \| 0 \| \| 0 \| \| 0 \| \| 0 \| \| 0 \| \| 1 \| \| 0 \| \| 0 \| \| 1 \| \| 1 \| \| 0 \| \| 0 \| \| 0 \| \| 0 \| \| 0 \| \| 0 \| \| 0 \| \| 0 \| \| 0 \| \| 0 \| \| 0 \| \| 0 \| \| 0 \| \| 0 \| \| 1 \| \| 0 \| \| 0 \| \| 0 \| \| 0 \| \| 1 \| \| 0 \| | \| 1098 \| \| --- \| \| 531 \| \| 66 \| \| 2177 \| \| 510 \| \| 183 \| \| 511 \| \| 865 \| \| 3002 \| \| 159 \| \| 84 \| \| 271 \| \| 385 \| \| 227 \| \| 262 \| \| 114 \| \| 1496 \| \| 873 \| \| 374 \| \| 316 \| \| 33 \| \| 4335 \| \| 164 \| \| 360 \| \| 201 \| \| 1580 \| \| 237 \| \| 13 \| \| 56 \| \| 910 \| \| 82 \| \| 704 \| \| 1065 \| \| 147 \| \| 147 \| \| 581 \| \| 869 \| \| 319 \| \| 621 \| \| 823 \| \| 896 \| \| 204 \| \| 746 \| \| 114 \| \| 157 \| \| 381 \| \| 1467 \| \| 27 \| \| 104 \| \| 147 \| \| 1958 \| \| 93 \| \| 26 \| \| 724 \| \| 349 \| \| 5812 \| \| 4185 \| \| 211 \| \| 68 \| \| 307 \| \| 549 \| \| 134 \| \| 2368 \| \| 255 \| \| 256 \| \| 383 \| \| 51 \| \| 1332 \| \| 431 \| \| 383 \| \| 293 \| \| 1470 \| \| 413 \| \| 319 \| \| 183 \| \| 71 \| \| 574 \| \| 9 \| \| 122 \| \| 322 \| \| 157 \| \| 461 \| \| 1587 \| \| 67 \| \| 541 \| \| 2607 \| \| 322 \| \| 316 \| \| 110 \| \| 2345 \| \| 615 \| \| 56 \| \| 55 \| \| 803 \| \| 267 \| \| 793 \| \| 66 \| \| 3435 \| \| 1314 \| \| 1599 \| \| 262 \| \| 1329 \| \| 529 \| \| 61 \| \| 2113 \| \| 208 \| \| 268 \| \| 5337 \| \| 290 \| \| 479 \| \| 1314 \| \| 4456 \| \| 75 \| \| 219 \| \| 569 \| \| 524 \| \| 12 \| \| 502 \| \| 356 \| \| 317 \| \| 320 \| \| 101 \| \| 300 \| \| 1254 \| \| 291 \| \| 183 \| \| 577 \| \| 577 \| \| 1857 \| \| 1108 \| \| 220 \| \| 7412 \| \| 179 \| \| 3857 \| \| 680 \| \| 99 \| \| 485 \| \| 212 \| \| 86 \| \| 583 \| \| 63 \| \| 152 \| \| 4284 \| \| 442 \| \| 120 \| \| 5409 \| \| 138 \| \| 1730 \| \| 308 \| \| 349 \| \| 147 \| \| 178 \| \| 170 \| \| 292 \| \| 445 \| \| 507 \| \| 864 \| \| 123 \| \| 1061 \| \| 103 \| \| 1913 \| \| 318 \| \| 583 \| \| 168 \| \| 9373 \| \| 536 \| \| 558 \| \| 599 \| \| 1304 \| \| 352 \| \| 184 \| \| 333 \| \| 2571 \| \| 611 \| \| 1726 \| \| 22 \| \| 3120 \| \| 211 \| \| 584 \| \| 150 \| \| 76 \| \| 1929 \| \| 96 \| \| 26 \| \| 81 \| \| 101 \| \| 55 \| \| 173 \| \| 46 \| \| 39 \| \| 1366 \| \| 172 \| \| 1038 \| \| 594 \| \| 189 \| \| 350 \| \| 223 \| \| 438 \| \| 1940 \| \| 935 \| \| 514 \| \| 53 \| \| 139 \| \| 1228 \| \| 7786 \| \| 603 \| \| 110 \| \| 401 \| \| 922 \| \| 18 \| \| 43 \| \| 97 \| \| 221 \| \| 559 \| \| 20 \| \| 636 \| \| 13 \| \| 3089 \| \| 70 \| \| 196 \| \| 98 \| \| 122 \| \| 2612 \| \| 194 \| \| 931 \| \| 53 \| \| 123 \| \| 27 \| \| 296 \| \| 26 \| \| 167 \| \| 5689 \| \| 302 \| \| 111 \| \| 29 \| \| 1429 \| \| 152 \| \| 148 \| \| 127 \| \| 404 \| \| 1258 \| \| 334 \| \| 1517 \| \| 147 \| \| 283 \| \| 134 \| \| 1914 \| \| 143 \| \| 158 \| \| 226 \| \| 757 \| \| 1024 \| \| 3727 \| \| 216 \| \| 242 \| \| 117 \| \| 451 \| \| 98 \| \| 153 \| \| 271 \| \| 127 \| \| 380 \| \| 272 \| \| 308 \| \| 158 \| \| 155 \| \| 295 \| \| 303 \| \| 376 \| \| 5627 \| \| 17 \| \| 60 \| \| 289 \| \| 93 \| \| 361 \| \| 347 \| \| 118 \| \| 154 \| \| 148 \| \| 1528 \| \| 543 \| \| 25 \| \| 28 \| \| 451 \| \| 2876 \| \| 28 \| \| 264 \| \| 153 \| \| 894 \| \| 472 \| \| 159 \| \| 189 \| \| 115 \| \| 95 \| \| 375 \| \| 36 \| \| 209 \| \| 146 \| \| 880 \| \| 23 \| \| 606 \| \| 869 \| \| 474 \| \| 24 \| \| 134 \| \| 2190 \| \| 521 \| \| 133 \| \| 227 \| \| 2446 \| \| 472 \| \| 418 \| \| 624 \| \| 380 \| \| 77 \| \| 2711 \| \| 50 \| \| 524 \| \| 125 \| \| 219 \| \| 151 \| \| 244 \| \| 1905 \| \| 17 \| \| 513 \| \| 664 \| \| 233 \| \| 126 \| \| 134 \| \| 123 \| \| 457 \| \| 98 \| \| 30 \| \| 17 \| \| 35 \| \| 45 \| \| 549 \| \| 260 \| \| 39 \| \| 168 \| \| 63 \| \| 171 \| \| 223 \| \| 50 \| \| 1362 \| \| 114 \| \| 3413 \| \| 28 \| \| 119 \| \| 86 \| \| 653 \| \| 74 \| \| 1975 \| \| 56 \| \| 67 \| \| 64 \| \| 350 \| \| 59 \| \| 46 \| \| 330 \| \| 27 \| \| 86 \| \| 18 \| \| 328 \| \| 14 \| \| 45 \| \| 9 \| \| 605 \| \| 180 \| \| 207 \| \| 112 \| \| 181 \| \| 74 \| \| 50 \| \| 50 \| \| 82 \| \| 57 \| \| 250 \| \| 320 \| \| 11085 \| \| 1279 \| \| 148 \| \| 106 \| \| 205 \| \| 382 \| \| 715 \| \| 257 \| \| 212 \| \| 131 \| \| 498 \| \| 149 \| \| 264 \| \| 392 \| \| 130 \| \| 203 \| \| 496 \| \| 144 \| \| 229 \| \| 223 \| \| 337 \| \| 240 \| \| 176 \| | \| 9000 \| \| --- \| \| 7638 \| \| 2814 \| \| 2803 \| \| 7544 \| \| 3682 \| \| 7500 \| \| 2449 \| \| 20609 \| \| 7559 \| \| 3075 \| \| 6716 \| \| 2203 \| \| 1128 \| \| 1657 \| \| 1016 \| \| 2519 \| \| 2031 \| \| 7541 \| \| 5459 \| \| 1425 \| \| 9000 \| \| 2719 \| \| 9000 \| \| 5351 \| \| 35000 \| \| 9000 \| \| 3673 \| \| 4302 \| \| 9000 \| \| 4095 \| \| 9000 \| \| 14815 \| \| 2210 \| \| 4115 \| \| 3902 \| \| 850 \| \| 31066 \| \| 2394 \| \| 9000 \| \| 808 \| \| 957 \| \| 3271 \| \| 2717 \| \| 9302 \| \| 9000 \| \| 9000 \| \| 750 \| \| 1606 \| \| 2446 \| \| 9000 \| \| 1111 \| \| 16577 \| \| 5207 \| \| 2849 \| \| 28160 \| \| 16663 \| \| 2988 \| \| 3385 \| \| 3710 \| \| 6449 \| \| 4610 \| \| 209 \| \| 9000 \| \| 17227 \| \| 1556 \| \| 4154 \| \| 1950 \| \| 1450 \| \| 9000 \| \| 5228 \| \| 9000 \| \| 2229 \| \| 1612 \| \| 9000 \| \| 4293 \| \| 15645 \| \| 5316 \| \| 1911 \| \| 4634 \| \| 3604 \| \| 1162 \| \| 1279 \| \| 4818 \| \| 16695 \| \| 13154 \| \| 6826 \| \| 2120 \| \| 1504 \| \| 33101 \| \| 2268 \| \| 5278 \| \| 3238 \| \| 6710 \| \| 750 \| \| 35000 \| \| 1826 \| \| 12659 \| \| 35000 \| \| 31484 \| \| 18846 \| \| 1750 \| \| 28783 \| \| 3922 \| \| 6500 \| \| 9000 \| \| 4213 \| \| 3632 \| \| 1858 \| \| 2323 \| \| 18369 \| \| 13691 \| \| 7702 \| \| 6869 \| \| 6144 \| \| 20770 \| \| 1263 \| \| 22459 \| \| 7399 \| \| 5904 \| \| 11534 \| \| 16414 \| \| 9000 \| \| 35000 \| \| 4552 \| \| 4959 \| \| 7913 \| \| 35000 \| \| 6860 \| \| 9000 \| \| 4255 \| \| 35000 \| \| 4822 \| \| 35000 \| \| 35000 \| \| 9000 \| \| 5715 \| \| 17731 \| \| 9000 \| \| 13037 \| \| 3088 \| \| 4243 \| \| 6183 \| \| 4461 \| \| 9476 \| \| 9000 \| \| 4322 \| \| 1863 \| \| 9000 \| \| 3676 \| \| 5593 \| \| 2468 \| \| 9000 \| \| 9000 \| \| 1483 \| \| 3798 \| \| 6894 \| \| 3273 \| \| 25731 \| \| 9000 \| \| 6682 \| \| 1057 \| \| 1370 \| \| 3339 \| \| 9000 \| \| 13017 \| \| 1620 \| \| 5125 \| \| 35000 \| \| 7500 \| \| 5296 \| \| 7778.5 \| \| 35000 \| \| 35000 \| \| 11080 \| \| 3750 \| \| 35000 \| \| 11809 \| \| 9162 \| \| 8657 \| \| 727 \| \| 9000 \| \| 3198 \| \| 2754 \| \| 7228 \| \| 10120 \| \| 8248 \| \| 9298 \| \| 7778.5 \| \| 9880 \| \| 4418 \| \| 6610 \| \| 5210 \| \| 4260 \| \| 7778.5 \| \| 9000 \| \| 1744 \| \| 9508 \| \| 35000 \| \| 7641 \| \| 9000 \| \| 2984 \| \| 7542 \| \| 2539 \| \| 35000 \| \| 78.5 \| \| 2651 \| \| 4147 \| \| 16828 \| \| 1693 \| \| 3892 \| \| 10836 \| \| 9000 \| \| 35000 \| \| 1047 \| \| 9000 \| \| 3070 \| \| 1647 \| \| 3112 \| \| 1228 \| \| 2696 \| \| 499.5 \| \| 35000 \| \| 960 \| \| 7229 \| \| 1984 \| \| 1221 \| \| 3697 \| \| 391 \| \| 4260 \| \| 4291 \| \| 11063 \| \| 2883 \| \| 1899 \| \| 4698 \| \| 35000 \| \| 1974 \| \| 6476 \| \| 13979 \| \| 5871 \| \| 9000 \| \| 9422 \| \| 6972 \| \| 7778.5 \| \| 7778.5 \| \| 766.6 \| \| 2658 \| \| 3680 \| \| 1799 \| \| 5233 \| \| 2870 \| \| 7228 \| \| 35000 \| \| 4153 \| \| 9379 \| \| 17527 \| \| 4422 \| \| 2637 \| \| 1044 \| \| 4680 \| \| 4065 \| \| 1919 \| \| 2280 \| \| 3968 \| \| 7927 \| \| 7778.5 \| \| 276.2 \| \| 6208 \| \| 6208 \| \| 6260 \| \| 168.3 \| \| 9000 \| \| 2045 \| \| 9000 \| \| 11039 \| \| 7229 \| \| 12604 \| \| 6979 \| \| 4787 \| \| 23211 \| \| 4549 \| \| 1558 \| \| 2243 \| \| 9000 \| \| 35000 \| \| 9000 \| \| 2967 \| \| 7356 \| \| 7778.5 \| \| 1276 \| \| 436.5 \| \| 1422 \| \| 1544 \| \| 3235 \| \| 3415 \| \| 7778.5 \| \| 1900 \| \| 84.32 \| \| 14458 \| \| 2912 \| \| 1664 \| \| 2849 \| \| 3734 \| \| 2213 \| \| 7189 \| \| 35000 \| \| 7291 \| \| 689.3 \| \| 1080 \| \| 9000 \| \| 7778.5 \| \| 13993 \| \| 1874 \| \| 9000 \| \| 6463 \| \| 9000 \| \| 2804 \| \| 4497 \| \| 5794 \| \| 2471 \| \| 1262 \| \| 9000 \| \| 9000 \| \| 7259 \| \| 11221 \| \| 168 \| \| 2887 \| \| 2507 \| \| 1924 \| \| 145 \| \| 6661 \| \| 1785 \| \| 7778.5 \| \| 1425 \| \| 9000 \| \| 2420 \| \| 11019 \| \| 35000 \| \| 7778.5 \| \| 1238 \| \| 226.5 \| \| 16470 \| \| 1363 \| \| 821.9 \| \| 17178 \| \| 3214 \| \| 21082 \| \| 9000 \| \| 200.2 \| \| 10677 \| \| 9000 \| \| 1604 \| \| 7758 \| \| 3311 \| \| 3576 \| \| 79.79 \| \| 9000 \| \| 9453 \| \| 2830 \| \| 1210 \| \| 1549 \| \| 7778.5 \| \| 9802 \| \| 1490 \| \| 6894 \| \| 9013 \| \| 306.7 \| \| 15306 \| \| 9000 \| \| 9000 \| \| 167.9 \| \| 1412 \| \| 1487 \| \| 11187 \| \| 1553 \| \| 183.9 \| \| 6531 \| \| 4621 \| \| 29194 \| \| 5880 \| \| 9000 \| \| 5286 \| \| 62.47 \| \| 9000 \| \| 1754 \| \| 690.1 \| \| 21103 \| \| 2349 \| \| 7898 \| \| 1469 \| \| 3090 \| \| 8835 \| \| 9000 \| \| 1986 \| \| 2798 \| \| 6303 \| \| 7778.5 \| \| 5322 \| \| 9891 \| \| 9000 \| \| 2489 \| \| 1622 \| |
| --- | --- | --- | --- | --- | --- | --- | --- | --- | --- | --- | --- | --- | --- | --- | --- | --- | --- | --- | --- | --- | --- | --- | --- | --- | --- | --- | --- | --- | --- | --- | --- | --- | --- | --- | --- | --- | --- | --- | --- | --- | --- | --- | --- | --- | --- | --- | --- | --- | --- | --- | --- | --- | --- | --- | --- | --- | --- | --- | --- | --- | --- | --- | --- | --- | --- | --- | --- | --- | --- | --- | --- | --- | --- | --- | --- | --- | --- | --- | --- | --- | --- | --- | --- | --- | --- | --- | --- | --- | --- | --- | --- | --- | --- | --- | --- | --- | --- | --- | --- | --- | --- | --- | --- | --- | --- | --- | --- | --- | --- | --- | --- | --- | --- | --- | --- | --- | --- | --- | --- | --- | --- | --- | --- | --- | --- | --- | --- | --- | --- | --- | --- | --- | --- | --- | --- | --- | --- | --- | --- | --- | --- | --- | --- | --- | --- | --- | --- | --- | --- | --- | --- | --- | --- | --- | --- | --- | --- | --- | --- | --- | --- | --- | --- | --- | --- | --- | --- | --- | --- | --- | --- | --- | --- | --- | --- | --- | --- | --- | --- | --- | --- | --- | --- | --- | --- | --- | --- | --- | --- | --- | --- | --- | --- | --- | --- | --- | --- | --- | --- | --- | --- | --- | --- | --- | --- | --- | --- | --- | --- | --- | --- | --- | --- | --- | --- | --- | --- | --- | --- | --- | --- | --- | --- | --- | --- | --- | --- | --- | --- | --- | --- | --- | --- | --- | --- | --- | --- | --- | --- | --- | --- | --- | --- | --- | --- | --- | --- | --- | --- | --- | --- | --- | --- | --- | --- | --- | --- | --- | --- | --- | --- | --- | --- | --- | --- | --- | --- | --- | --- | --- | --- | --- | --- | --- | --- | --- | --- | --- | --- | --- | --- | --- | --- | --- | --- | --- | --- | --- | --- | --- | --- | --- | --- | --- | --- | --- | --- | --- | --- | --- | --- | --- | --- | --- | --- | --- | --- | --- | --- | --- | --- | --- | --- | --- | --- | --- | --- | --- | --- | --- | --- | --- | --- | --- | --- | --- | --- | --- | --- | --- | --- | --- | --- | --- | --- | --- | --- | --- | --- | --- | --- | --- | --- | --- | --- | --- | --- | --- | --- | --- | --- | --- | --- | --- | --- | --- | --- | --- | --- | --- | --- | --- | --- | --- | --- | --- | --- | --- | --- | --- | --- | --- | --- | --- | --- | --- | --- | --- | --- | --- | --- | --- | --- | --- | --- | --- | --- | --- | --- | --- | --- | --- | --- | --- | --- | --- | --- | --- | --- | --- | --- | --- | --- | --- | --- | --- | --- | --- | --- | --- | --- | --- | --- | --- | --- | --- | --- | --- | --- | --- | --- | --- | --- | --- | --- | --- | --- | --- | --- | --- | --- | --- | --- | --- | --- | --- | --- | --- | --- | --- | --- | --- | --- | --- | --- | --- | --- | --- | --- | --- | --- | --- | --- | --- | --- | --- | --- | --- | --- | --- | --- | --- | --- | --- | --- | --- | --- | --- | --- | --- | --- | --- | --- | --- | --- | --- | --- | --- | --- | --- | --- | --- | --- | --- | --- | --- | --- | --- | --- | --- | --- | --- | --- | --- | --- | --- | --- | --- | --- | --- | --- | --- | --- | --- | --- | --- | --- | --- | --- | --- | --- | --- | --- | --- | --- | --- | --- | --- | --- | --- | --- | --- | --- | --- | --- | --- | --- | --- | --- | --- | --- | --- | --- | --- | --- | --- | --- | --- | --- | --- | --- | --- | --- | --- | --- | --- | --- | --- | --- | --- | --- | --- | --- | --- | --- | --- | --- | --- | --- | --- | --- | --- | --- | --- | --- | --- | --- | --- | --- | --- | --- | --- | --- | --- | --- | --- | --- | --- | --- | --- | --- | --- | --- | --- | --- | --- | --- | --- | --- | --- | --- | --- | --- | --- | --- | --- | --- | --- | --- | --- | --- | --- | --- | --- | --- | --- | --- | --- | --- | --- | --- | --- | --- | --- | --- | --- | --- | --- | --- | --- | --- | --- | --- | --- | --- | --- | --- | --- | --- | --- | --- | --- | --- | --- | --- | --- | --- | --- | --- | --- | --- | --- | --- | --- | --- | --- | --- | --- | --- | --- | --- | --- | --- | --- | --- | --- | --- | --- | --- | --- | --- | --- | --- | --- | --- | --- | --- | --- | --- | --- | --- | --- | --- | --- | --- | --- | --- | --- | --- | --- | --- | --- | --- | --- | --- | --- | --- | --- | --- | --- | --- | --- | --- | --- | --- | --- | --- | --- | --- | --- | --- | --- | --- | --- | --- | --- | --- | --- | --- | --- | --- | --- | --- | --- | --- | --- | --- | --- | --- | --- | --- | --- | --- | --- | --- | --- | --- | --- | --- | --- | --- | --- | --- | --- | --- | --- | --- | --- | --- | --- | --- | --- | --- | --- | --- | --- | --- | --- | --- | --- | --- | --- | --- | --- | --- | --- | --- | --- | --- | --- | --- | --- | --- | --- | --- | --- | --- | --- | --- | --- | --- | --- | --- | --- | --- | --- | --- | --- | --- | --- | --- | --- | --- | --- | --- | --- | --- | --- | --- | --- | --- | --- | --- | --- | --- | --- | --- | --- | --- | --- | --- | --- | --- | --- | --- | --- | --- | --- | --- | --- | --- | --- | --- | --- | --- | --- | --- | --- | --- | --- | --- | --- | --- | --- | --- | --- | --- | --- | --- | --- | --- | --- | --- | --- | --- | --- | --- | --- | --- | --- | --- | --- | --- | --- | --- | --- | --- | --- | --- | --- | --- | --- | --- | --- | --- | --- | --- | --- | --- | --- | --- | --- | --- | --- | --- | --- | --- | --- | --- | --- | --- | --- | --- | --- | --- | --- | --- | --- | --- | --- | --- | --- | --- | --- | --- | --- | --- | --- | --- | --- | --- | --- | --- | --- | --- | --- | --- | --- | --- | --- | --- | --- | --- | --- | --- | --- | --- | --- | --- | --- | --- | --- | --- | --- | --- | --- | --- | --- | --- | --- | --- | --- | --- | --- | --- | --- | --- | --- | --- | --- | --- | --- | --- | --- | --- | --- | --- | --- | --- | --- | --- | --- | --- | --- | --- | --- | --- | --- | --- | --- | --- | --- | --- | --- | --- | --- | --- | --- | --- | --- | --- | --- | --- | --- | --- | --- | --- | --- | --- | --- | --- | --- | --- | --- | --- | --- | --- | --- | --- | --- | --- | --- | --- | --- | --- | --- | --- | --- | --- | --- | --- | --- | --- | --- | --- | --- | --- | --- | --- | --- | --- | --- | --- | --- | --- | --- | --- | --- | --- | --- | --- | --- | --- | --- | --- | --- | --- | --- | --- | --- | --- | --- | --- | --- | --- | --- | --- | --- | --- | --- | --- | --- | --- | --- | --- | --- | --- | --- | --- | --- | --- | --- | --- | --- | --- | --- | --- | --- | --- | --- | --- | --- | --- | --- | --- | --- | --- | --- | --- | --- | --- | --- | --- | --- | --- | --- | --- | --- | --- | --- | --- | --- | --- | --- | --- | --- | --- | --- | --- | --- | --- | --- | --- | --- | --- | --- | --- | --- | --- | --- | --- | --- | --- | --- | --- | --- | --- | --- | --- | --- | --- | --- | --- | --- | --- | --- | --- | --- | --- | --- | --- | --- | --- | --- | --- | --- | --- | --- | --- | --- | --- | --- | --- | --- | --- | --- | --- | --- | --- | --- | --- | --- | --- | --- | --- | --- | --- | --- | --- | --- | --- | --- | --- | --- | --- | --- | --- | --- | --- | --- | --- | --- | --- | --- | --- | --- | --- | --- | --- | --- | --- | --- | --- | --- | --- | --- | --- | --- | --- | --- | --- | --- | --- | --- | --- | --- | --- | --- | --- | --- | --- | --- | --- | --- | --- | --- | --- | --- | --- | --- | --- | --- | --- | --- | --- | --- | --- | --- | --- | --- | --- | --- | --- | --- | --- | --- | --- | --- | --- | --- | --- | --- | --- | --- | --- | --- | --- | --- | --- | --- | --- | --- | --- | --- | --- | --- | --- | --- | --- | --- | --- | --- | --- | --- | --- | --- | --- | --- | --- | --- | --- | --- | --- | --- | --- | --- | --- | --- | --- | --- | --- | --- | --- | --- | --- | --- | --- | --- | --- | --- | --- | --- | --- | --- | --- | --- | --- | --- | --- | --- | --- | --- | --- | --- | --- | --- | --- | --- | --- | --- | --- | --- | --- | --- | --- | --- | --- | --- | --- | --- | --- | --- | --- | --- | --- | --- | --- | --- | --- | --- | --- | --- | --- | --- | --- | --- | --- | --- | --- | --- | --- | --- | --- | --- | --- | --- | --- | --- | --- | --- | --- | --- | --- | --- | --- | --- | --- | --- | --- | --- | --- | --- | --- | --- | --- | --- | --- | --- | --- | --- | --- | --- | --- | --- | --- | --- | --- | --- | --- | --- | --- | --- | --- | --- | --- | --- | --- | --- | --- | --- | --- | --- | --- | --- | --- | --- | --- | --- | --- | --- | --- | --- | --- | --- | --- | --- | --- | --- | --- | --- | --- | --- | --- | --- | --- | --- | --- | --- | --- | --- | --- | --- | --- | --- | --- | --- | --- | --- | --- | --- | --- | --- | --- | --- | --- | --- | --- | --- | --- | --- | --- | --- | --- | --- | --- | --- | --- | --- | --- | --- | --- | --- | --- | --- | --- | --- | --- | --- | --- | --- | --- | --- | --- | --- | --- | --- | --- | --- | --- | --- | --- | --- | --- | --- | --- | --- | --- | --- | --- | --- | --- | --- | --- | --- | --- | --- | --- | --- | --- | --- | --- | --- | --- | --- | --- | --- | --- | --- | --- | --- | --- | --- | --- | --- | --- | --- | --- | --- | --- | --- | --- | --- | --- | --- | --- | --- | --- | --- | --- | --- | --- | --- | --- | --- | --- | --- | --- | --- | --- | --- | --- | --- | --- | --- | --- | --- | --- | --- | --- | --- | --- | --- | --- | --- | --- | --- | --- | --- | --- | --- | --- | --- | --- | --- | --- | --- | --- | --- | --- | --- | --- | --- | --- | --- | --- | --- | --- | --- | --- | --- | --- | --- | --- | --- | --- | --- | --- | --- | --- | --- | --- | --- | --- | --- | --- | --- | --- | --- | --- | --- | --- | --- | --- | --- | --- | --- | --- | --- | --- | --- | --- | --- | --- | --- | --- | --- | --- | --- | --- | --- | --- | --- | --- | --- | --- | --- | --- | --- | --- | --- | --- | --- | --- | --- | --- | --- | --- | --- | --- | --- | --- | --- | --- | --- | --- | --- | --- | --- | --- | --- | --- | --- | --- | --- | --- | --- | --- | --- | --- | --- | --- | --- | --- | --- | --- | --- | --- | --- | --- | --- | --- | --- | --- | --- | --- | --- | --- | --- | --- | --- | --- | --- | --- | --- | --- | --- | --- | --- | --- | --- | --- | --- | --- | --- | --- | --- | --- | --- | --- | --- | --- | --- | --- | --- | --- | --- | --- | --- | --- | --- | --- | --- | --- | --- | --- | --- | --- | --- | --- | --- | --- | --- | --- | --- | --- | --- | --- | --- | --- | --- | --- | --- | --- | --- | --- | --- | --- | --- | --- | --- | --- | --- | --- | --- | --- | --- | --- | --- | --- | --- | --- | --- | --- | --- | --- | --- | --- | --- | --- | --- | --- | --- | --- | --- | --- | --- | --- | --- | --- | --- | --- | --- | --- | --- | --- | --- | --- | --- | --- | --- | --- | --- | --- | --- | --- | --- | --- | --- | --- | --- | --- | --- | --- | --- | --- | --- | --- | --- | --- | --- | --- | --- | --- | --- | --- | --- | --- | --- | --- | --- | --- | --- | --- | --- | --- | --- | --- | --- | --- | --- | --- | --- | --- | --- | --- | --- | --- | --- | --- | --- | --- | --- | --- | --- | --- | --- | --- | --- | --- | --- | --- | --- | --- | --- | --- | --- | --- | --- | --- | --- | --- | --- | --- | --- | --- | --- | --- | --- | --- | --- | --- | --- | --- | --- | --- | --- | --- | --- | --- | --- | --- | --- | --- | --- | --- | --- | --- | --- | --- | --- | --- | --- | --- | --- | --- | --- | --- | --- | --- | --- | --- | --- | --- | --- | --- | --- | --- | --- | --- | --- | --- | --- | --- | --- | --- | --- | --- | --- | --- | --- | --- | --- | --- | --- | --- | --- | --- | --- | --- | --- | --- | --- | --- | --- | --- | --- | --- | --- | --- | --- | --- | --- | --- | --- | --- | --- | --- | --- | --- | --- | --- | --- | --- | --- | --- | --- | --- | --- | --- | --- | --- | --- | --- | --- | --- | --- | --- | --- | --- | --- | --- | --- | --- | --- | --- | --- | --- | --- | --- | --- | --- | --- | --- | --- | --- | --- | --- | --- | --- | --- | --- | --- | --- | --- | --- | --- | --- | --- | --- | --- | --- | --- | --- | --- | --- | --- | --- | --- | --- | --- | --- | --- | --- | --- | --- | --- | --- | --- | --- | --- | --- | --- | --- | --- | --- | --- | --- | --- | --- | --- | --- | --- | --- | --- | --- | --- | --- | --- | --- | --- | --- | --- | --- | --- | --- | --- | --- | --- | --- | --- | --- | --- | --- | --- | --- | --- | --- | --- | --- | --- | --- | --- | --- | --- | --- | --- |

The supporting information file of this paper contains 1 file, named “S1 Dataset”. In this file, we listed all the raw data from 402 AHF patients (72 patients was death during the follow-up). The statistic items include gender (1 expresses male and 2 expresses female), age. Others are clinical situation and the concentration of FGF21 (pg/ml) and NT-proBNP (pg/ml), such as a patient with (marked as 1) or without (marked as 0) coronary heart disease, diabetes mellitus, all-cause death.
